# Supplementary material for: GWAS of mosaic loss of chromosome Y highlights genetic effects on blood cell differentiation
Source: Nat Commun. 2019 Oct 17;10:4719. doi: 10.1038/s41467-019-12705-5 (PMC6797717; doi:10.1038/s41467-019-12705-5)
Supplement: Supplementary file 1 — Supplementary Information [file 41467_2019_12705_MOESM1_ESM.pdf]

Supplementary Materials for
Terao et al, GWAS of mosaic loss of chromosome Y highlights genetic effects on blood cell differentiation

Supplementary Note 1-8. Page 2-9
Supplementary Figure 1-15. Page 10-33
Supplementary Table 1-11. Page 34-50
Reference for Supplementary Materials. Page 50-51

Supplementary Note 1. Construction of a reference panel containing deep-depth whole-genome sequence data in the Japanese.

We constructed a reference panel for imputation by combining 2,504 1kg data (all populations) and deep-depth whole-genome sequence (WGS) data (x30) of 1,037 Japanese subjects. Data production of the WGS data was previously described in detail[1]. Briefly, WGS was conducted using the Hiseq2500 platform with the use of 160-bp paired-end reads. The standard method based on best practice in GATK (URL) was adopted to process the data. In addition to the process of the best practice, we put additional filters of approximate read depth (DP) and genotype quality (GQ) ((1) DP < 5, (2) GQ < 20, or (3) DP > 60 and GQ < 95) before variant quality score recalibration. We combined the two data sets separately for autosomes and X chromosomes as follows.

1. Autosome

After exclusion of variants at multi-allelic sites and singletons from the two data sets by bcftools or vcftools, IMPUTE2[2] (-merge_ref_panels) was used to merge the data sets by imputing variants not contained in either data set by referring the other data set. We used shapeit program[3] to phase the Japanese WGS data.

2. X chromosome

Pseudo autosomal region (PAR) and low complexity region (LCR) defined by mdust software (‘hs37d5-LCRs.20140224.bed’, see URL) were excluded. Since –panel-merge option in IMPUTE2 cannot be used for haploids, we used beagle4.1 software for males to impute variants missing in either of the data sets. Reference panel of males and females were combined with the use of GATK (-T Combine Variants), and we excluded variants 1) showing departure of Hardy-Weinberg disequilibrium with p-values less than 1.0×10^{-6} in females in the WGS, 2) with difference of allele frequency more than 5% between males and females in WGS, 3) in multi-allelic sites, 4) singletons.

The accuracy of imputation based on this reference panel is described in detail elsewhere (Akiyama et al, in press[4]).

Supplementary Note 2. Difference in lambda GC between LDSC regression and nominal associations.

Since LDSC took variants whose genotypes were highly accurate (in the current analyses, we adopted variants in the hapmap3 project) to ensure accurate genotypes, we observed slight difference in lambda GC between LDSC regression and nominal associations (1.086 and 1.066, respectively).

Supplementary Note 3. Associations with the use of other annotations for LD score regression.

We also applied to the analyses another full baseline model of ldsc recently reported including information of LD-related annotations, synonymous and non-synonymous annotations, ancient sequence age, and conserved function across species[5].

We confirmed the findings in the main text with the use of the latest full baseline model. Superenhancer showed the strongest enrichment in heritability for mLOY (2.8 times, $p=4.1\times10^{-5}$, p-value for enrichment). We also confirmed that histone marks associated with the hematopoietic cell group ($p=2.9\times10^{-8}$, p-value for enrichment) and CD34 primary cells ($p=1.7\times10^{-9}$, p-value for enrichment) demonstrated strong enrichment of mLOY heritability. For LDSC-SEG, MPP

and HSC showed the strongest and 2nd strongest heritability enrichment of mLOY associations among the cell types analyzed ($p=3.7x10^{-6}$ and $1.6x10^{-5}$ for MPP and HSC, respectively, p-values for enrichment). Binding sites of FLI1 also showed a strong enrichment of mLOY heritability ($p=9.0x10^{-7}$, p-value for enrichment) which was comparable with that using the full baseline model described in Finucane et al, 2015[6].

Supplementary Note 4. Genetic correlation between mLOY and quantitative trait or malignancy.

To study possible common mechanisms between mLOY and other quantitative trait loci, we conducted genetic correlation analysis with the use of results of 44 Japanese QTL data of blood measurements (JENGER, see URL). Overall, we did not identify prominent genetic correlation, which implies that these laboratory tests are independent of mLOY except for the positive correlation of aspartate transaminase (AST) (p=0.00020). A bidirectional Mendelian randomization approach suggests a causal effect of mLOY on increase of AST (p=0.037, Supplementary Figure 12), but not vice versa (p=0.77). Hematological traits did not show strong genetic correlations with mLOY (Supplementary Table 9). We

also assessed genetic correlation between susceptibility to malignancy and mLOY. We used summary statistics for a total of 13 cancer risk associations from a previous report[7]. None of them showed significant genetic correlation with mLOY (Supplementary Figure 15 and Supplementary Table 9). We also found that directions of the correlations (positive or negative) between mLOY and specific cancer types do not seem to be consistent across cancers.

Supplementary Note 5. Potential poor outcome in subjects with a high fraction of mLOY

Forceberg et al analyzed 982 elderly subjects without cancer history and showed mLOY was associated with poor survival and increased cancer onset[8]. They treated cell fractions with mLOY as a quantitative trait or binary trait with cut-off level of 35% of cells with mLOY (27 over 1,141 participants including subjects with cancer history) and showed the associations. The distribution of cell fraction with mLOY did not follow a normal distribution and the majority of subjects had cell fraction of less than 0.18. Thus, the significant associations seemed to be brought about by a subset of individuals with a high cell fraction of mLOY.

We hypothesized that subjects with a high cell fraction of mLOY might have poor outcome. To test the hypothesis, we took the top 3, 5, 10 and 20% of subjects with high mLOY signals in our data and analyzed survival in cox proportional hazards regression.

Supplementary Note 6. Evaluation of associations between mLOY and lung cancer mortality.

We found a borderline significant association between mLOY and lung cancer mortality, and so we carefully evaluated the association. First, we extracted smokers with information of smoking year and quantity of smoking (37% of smokers). We found that the effect size of mLOY was decreased and that the association was no longer significant (HR:1.04 (95%CI:0.96-1.13), p=0.37, Cox proportional hazards regression). Second, we extracted non-smokers and analyzed the association. We found that the direction in effect size of mLOY on lung cancer mortality in non-smokers was opposite to that of smokers (coefficient -0.044, p-value 0.71, Cox proportional hazards regression). Based on these findings, we should carefully assess the association and regard as inconclusive.

Supplementary Note 7. Difference in susceptibility loci between median and mean LRR-Y

We used the mean of LRR as a proxy of chromosome Y copy number since the previous study, Wright et al, 2017[9], used the similar approach. However, using the mean, rather than the median, of LRR allows that some strongly deviated markers could led to imprecise estimation of chromosome Y dosage. On this point, we found that the median and mean are strongly correlated (Spearman's rho:0.993) and lead to quite similar genetic association results. Genetic associations using median LRR-Y identifies 44 of the 46 loci identified using mean LRR-Y. The 2 loci significant in mean LRR-Y but not significant in median LRR-Y were *RBPMS* (a lead variant:rs2979469) on chromosome 8 and *KANK1* ((a lead variant:rs2804301) on chromosome 9. These two loci showed boundary associations in median LRR-Y ($p=6.9 \times 10^{-8}$ and 1.0×10^{-7}, bolt-lmm), reflecting the strong correlation between mean and median LRR-Y. These indicate that our approach results in robust associations and difference in associations between mean and median LRR-Y is minimal.

Supplementary Note 8. Deviation of disease distributions among the three data sets in the BBJ.

We found that more than 60% of subjects in the 3rd data set had malignancies in contrast to the other two data sets (less than 10%, Supplementary Table 1). This is because genotyping schedule of subjects in the BBJ was decided based on disease set-basis, such as patients with esophagus cancer. We put disease status and data sets as covariates and found a consistent association trend across the three data sets (Supplementary Table 2).

Supplementary Figure 1. Associations between mLOY and age at blood collection in the three data sets.

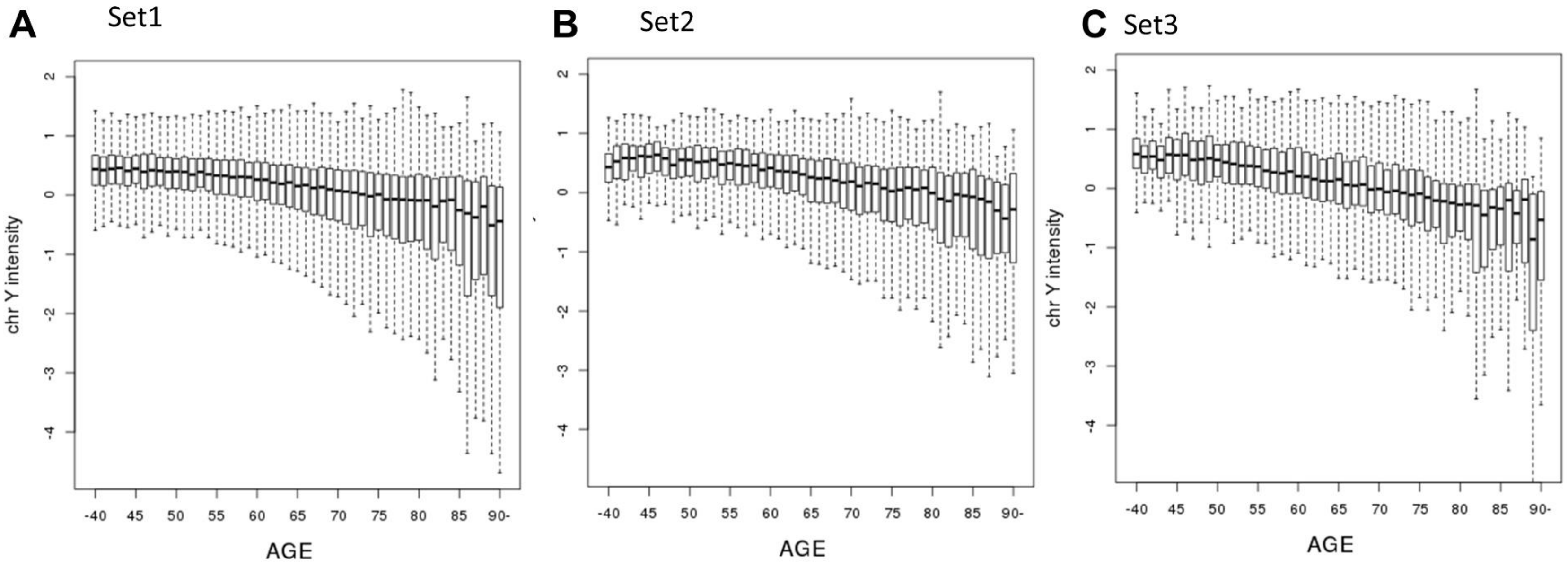

Associations between chr Y intensity and age at blood collection are indicated in A. set 1 (Illumina Human Omni Express Exome v1.0) B. set 2 (Illumina Human Omni Express Exome v1.2) and C. set 3 (Illumina Human Omni Express), respectively. Subjects with age under 40 and over 90 are grouped separately. We do not show outliers in the figure. Bars indicate the most extreme data points which are no more than 1.5 times interquartile ranges from the boxes.

Supplementary Figure 2. Lower chromosome Y intensity in smokers.

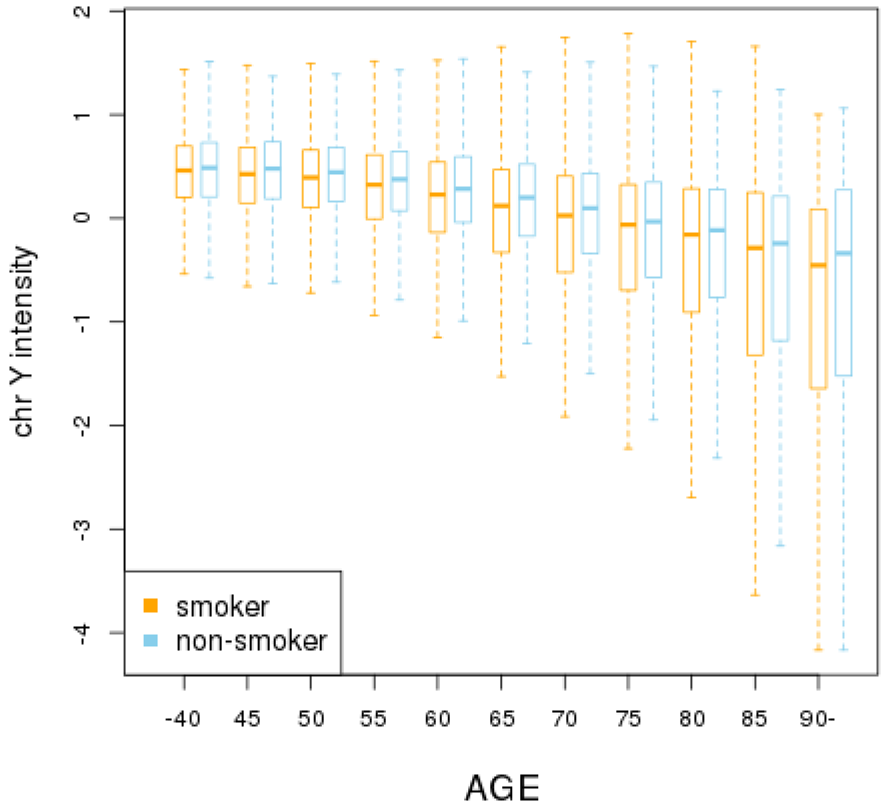

A distribution of chromosome Y intensity in smokers and non-smokers are indicated according to their age at blood collection. We do not show outliers in the figure. Bars indicate the most extreme data points which are no more than 1.5 times interquartile ranges from the boxes.

Supplementary Figure 3. Quantile-quantile plot of mLOY.

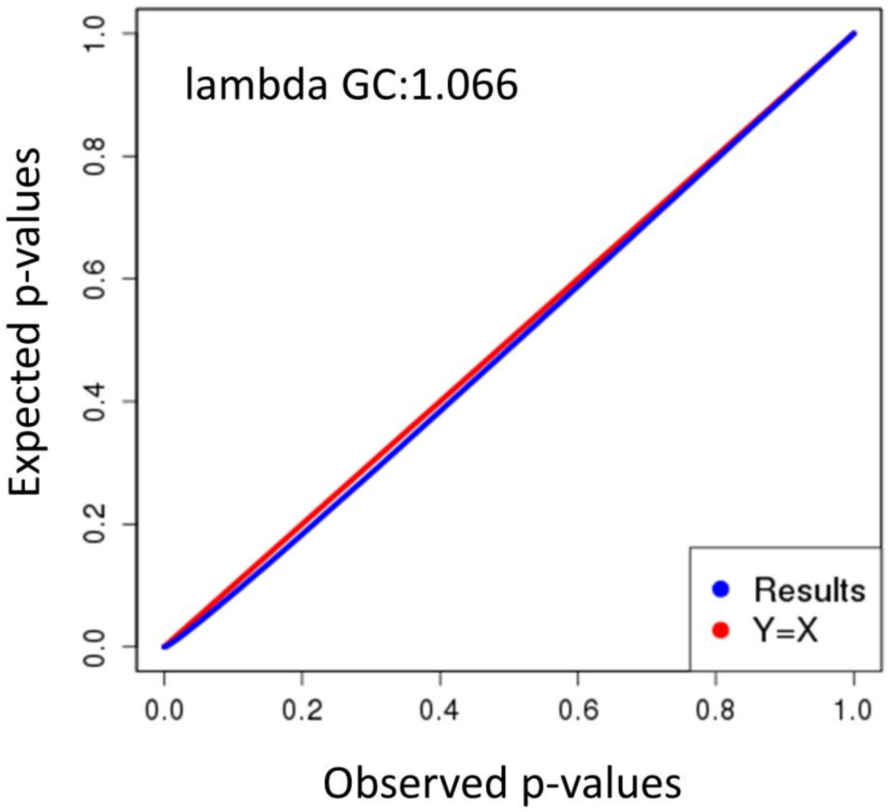

Observed and expected p-values in mLOY are indicated in X and Y axis, respectively, in quantile–quantile plot. Since this figure is designed to show the presence of systemic deviation of statistics, X and Y are not log transformed. Blue and red lines indicate p-values of observed and expected results, respectively.

Supplementary Figure 4. Regional plots of the unreported 31 loci for mLOY.

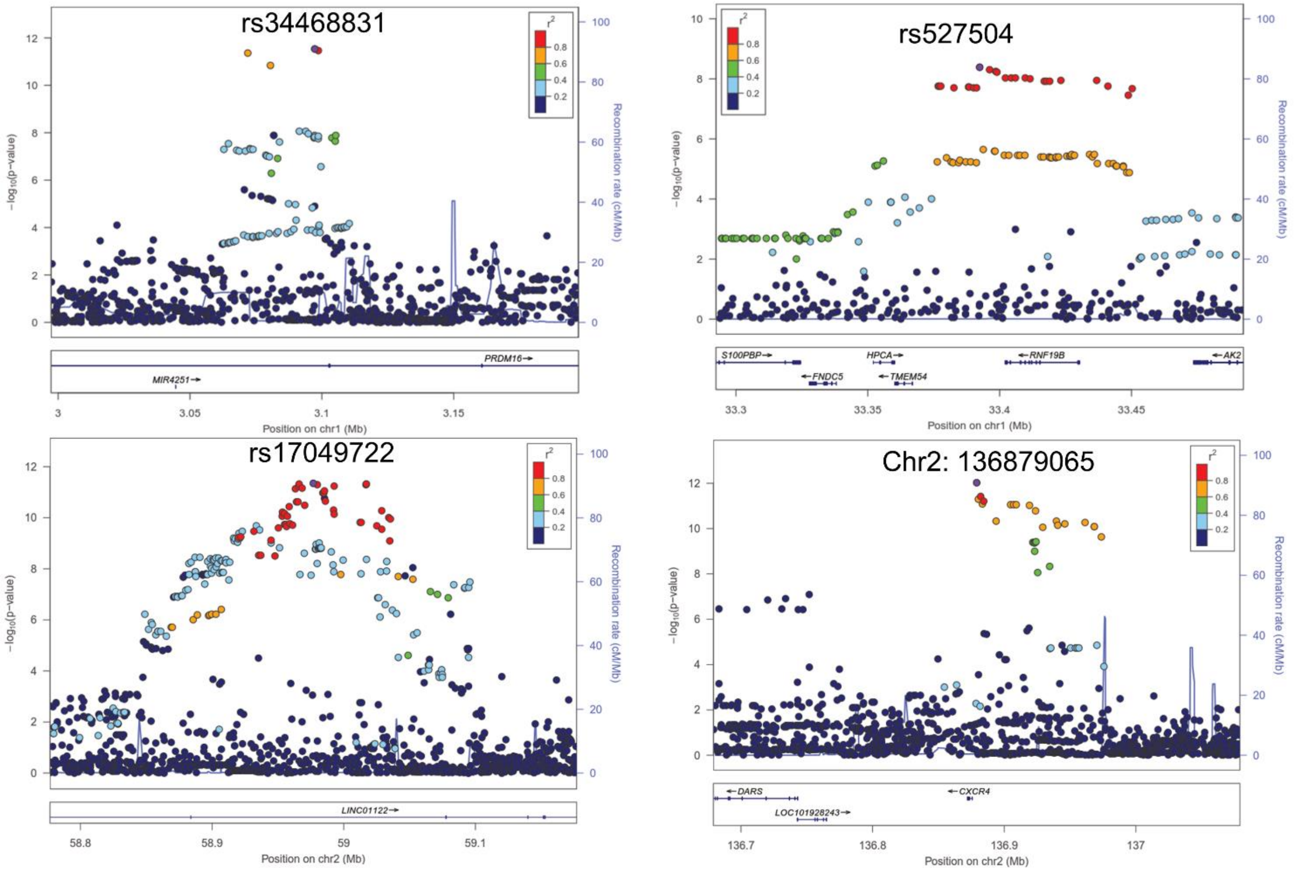

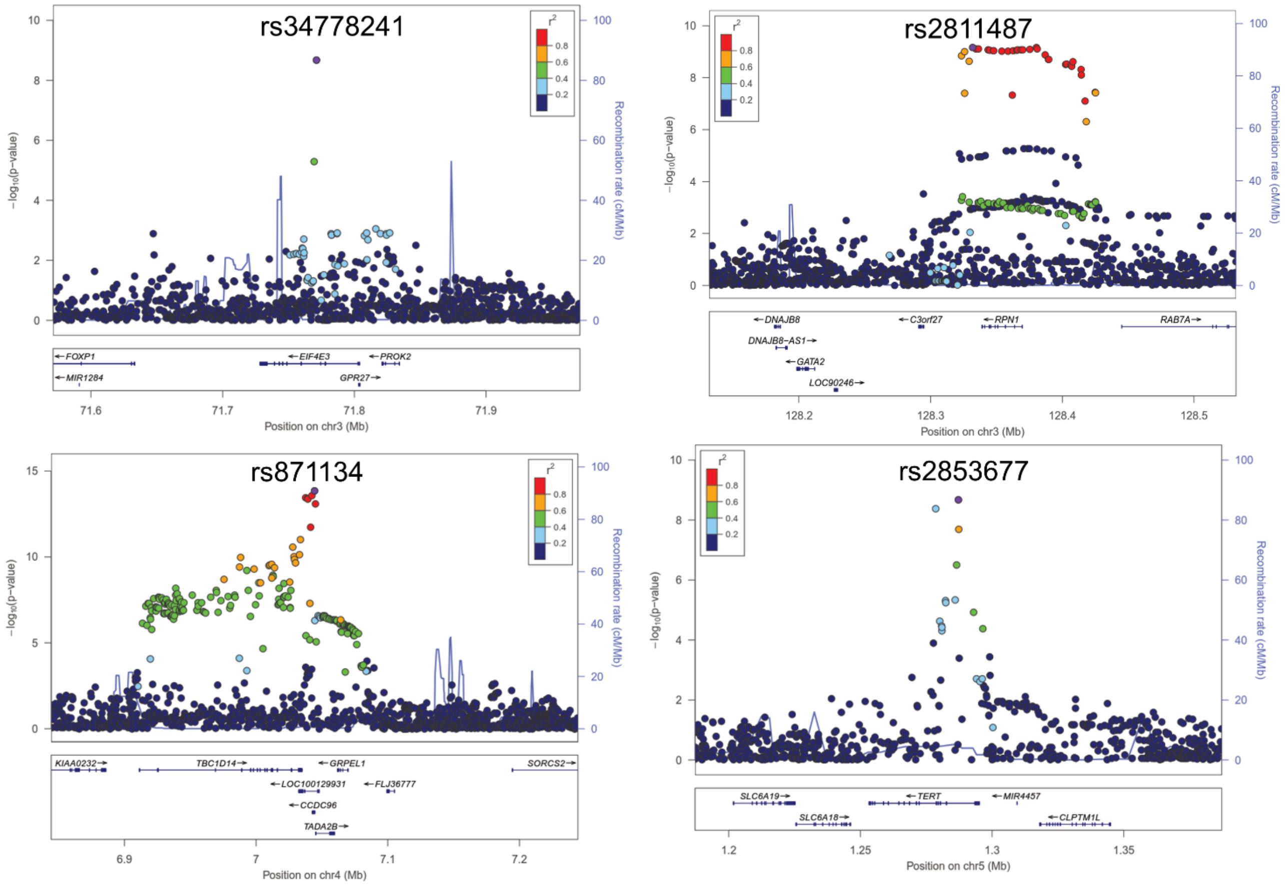
rs34778241
rs2811487
rs871134
rs2853677
r²
−log₁₀(p−value)
Recombination rate (cM/Mb)
FOXP1
MIR1284
EIF4E3
PROK2
GPR27
Position on chr3 (Mb)
DNAJB8
DNAJB8−AS1
GATA2
LOC90246
C3orf27
RPN1
RAB7A
Position on chr3 (Mb)
KIAA0232
TBC1D14
GRPEL1
SORCS2
LOC100129931
FLJ36777
CCDC96
TADA2B
Position on chr4 (Mb)
SLC6A19
SLC6A18
TERT
MIR4457
CLPTM1L
Position on chr5 (Mb)

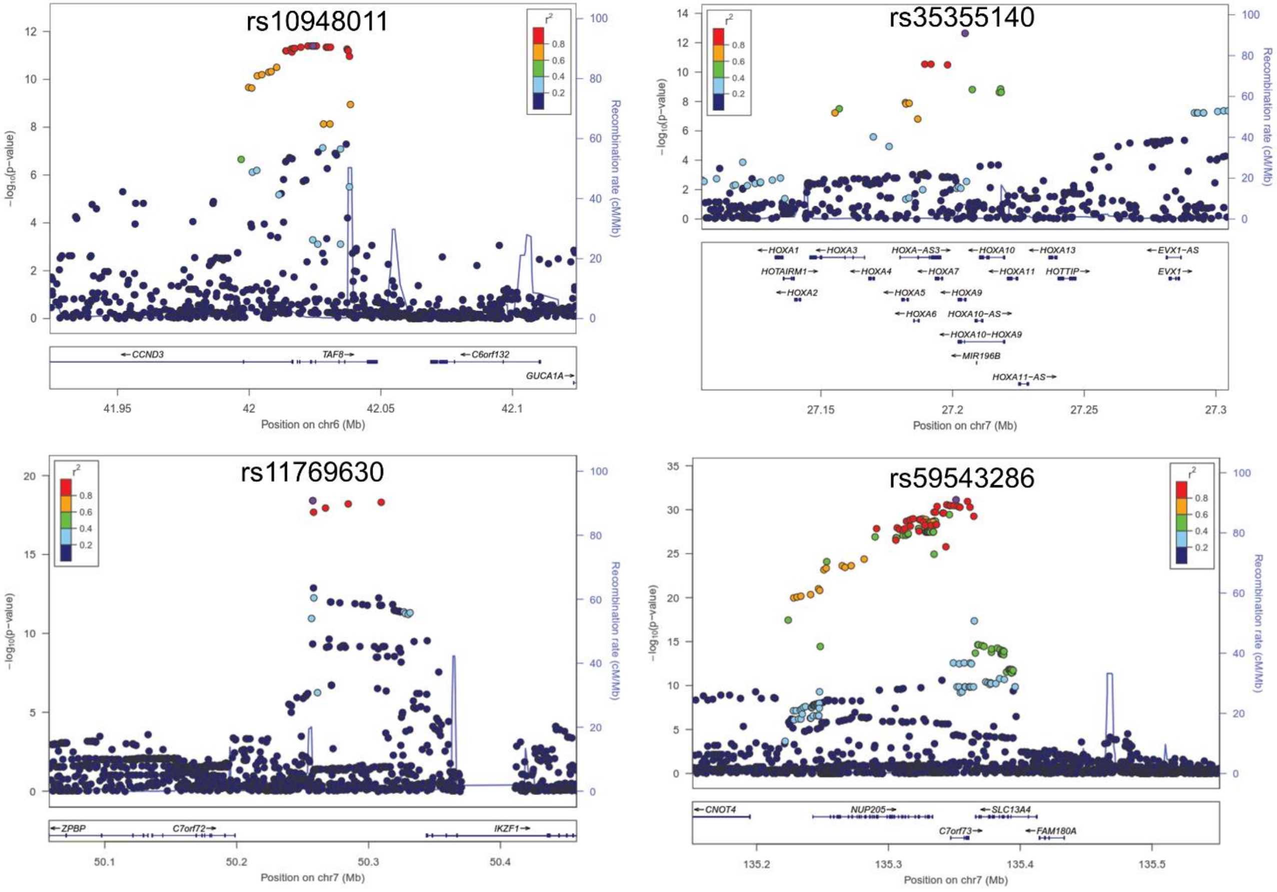
rs10948011
rs35355140
rs11769630
rs59543286
−log10(p−value)
Recombination rate (cM/Mb)
r²
CCND3
TAF8
C6orf132
GUCA1A
Position on chr6 (Mb)
HOXA1
HOXA3
HOXA−AS3
HOXA10
HOXA13
EVX1−AS
HOTAIRM1
HOXA4
HOXA7
HOXA11
HOTTIP
EVX1
HOXA2
HOXA5
HOXA9
HOXA6
HOXA10−AS
HOXA10−HOXA9
MIR196B
HOXA11−AS
Position on chr7 (Mb)
ZPBP
C7orf72
IKZF1
Position on chr7 (Mb)
CNOT4
NUP205
SLC13A4
C7orf73
FAM180A
Position on chr7 (Mb)

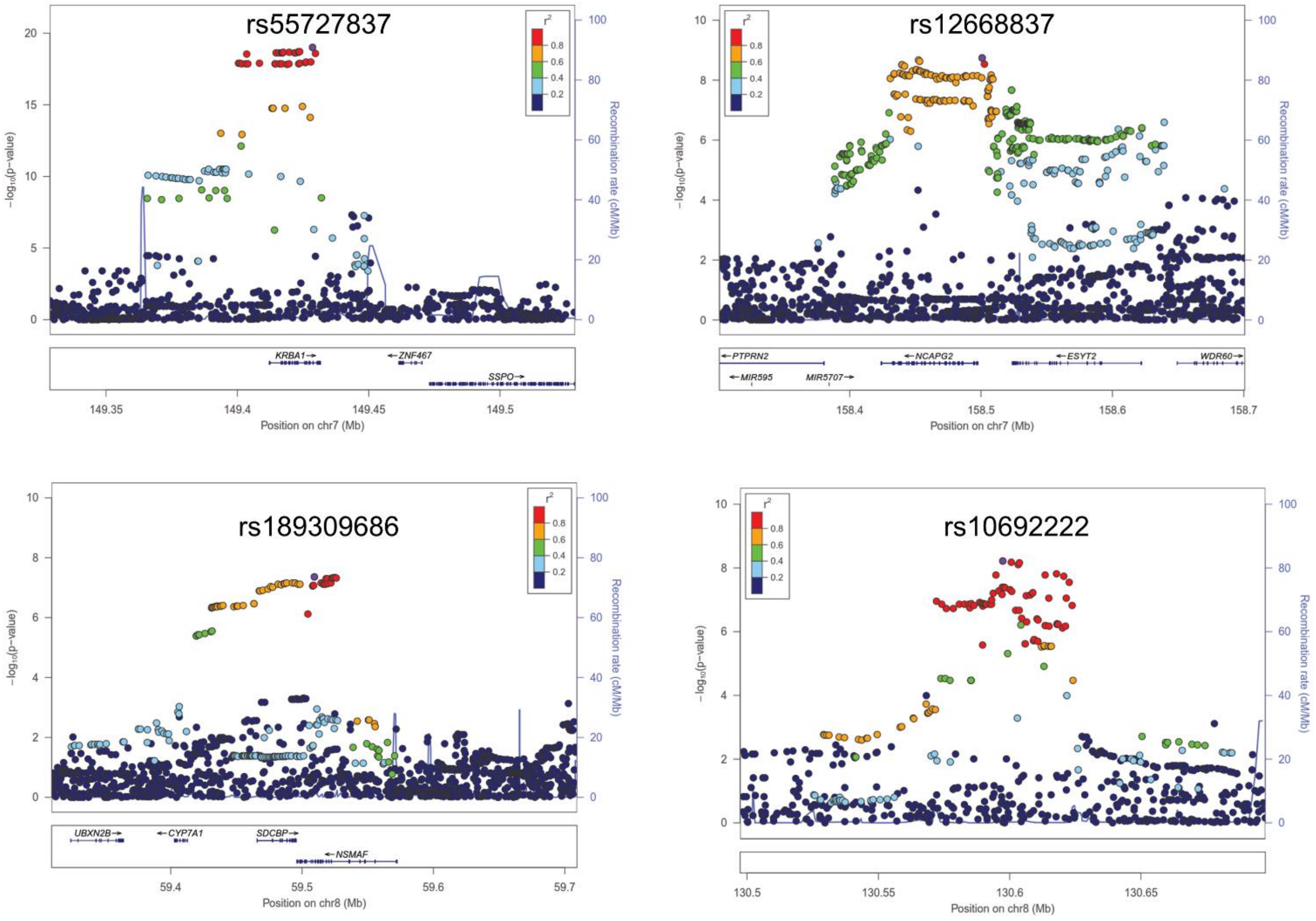
rs55727837
−log10(p−value)
Recombination rate (cM/Mb)
KRBA1
ZNF467
SSPO
Position on chr7 (Mb)
rs12668837
PTPRN2
MIR595
MIR5707
NCAPG2
ESYT2
WDR60
Position on chr7 (Mb)
rs189309686
UBXN2B
CYP7A1
SDCBP
NSMAF
Position on chr8 (Mb)
rs10692222
Position on chr8 (Mb)

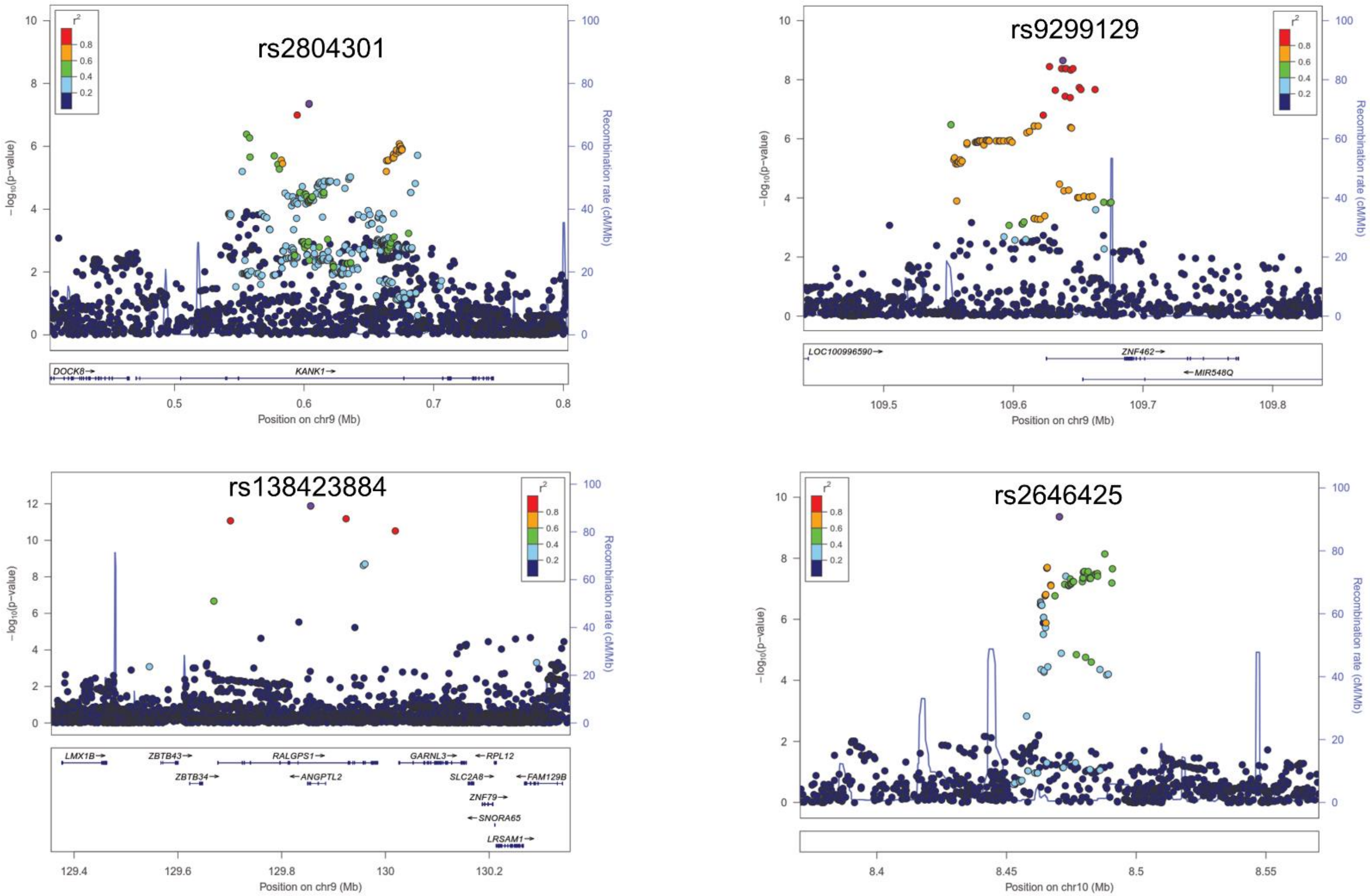
rs2804301
rs9299129
rs138423884
rs2646425
r²
0.8
0.6
0.4
0.2
−log₁₀(p−value)
Recombination rate (cM/Mb)
DOCK8
KANK1
Position on chr9 (Mb)
LOC100996590
ZNF462
MIR548Q
LMX1B
ZBTB43
ZBTB34
RALGPS1
ANGPTL2
GARNL3
RPL12
SLC2A8
FAM129B
ZNF79
SNORA65
LRSAM1
Position on chr10 (Mb)

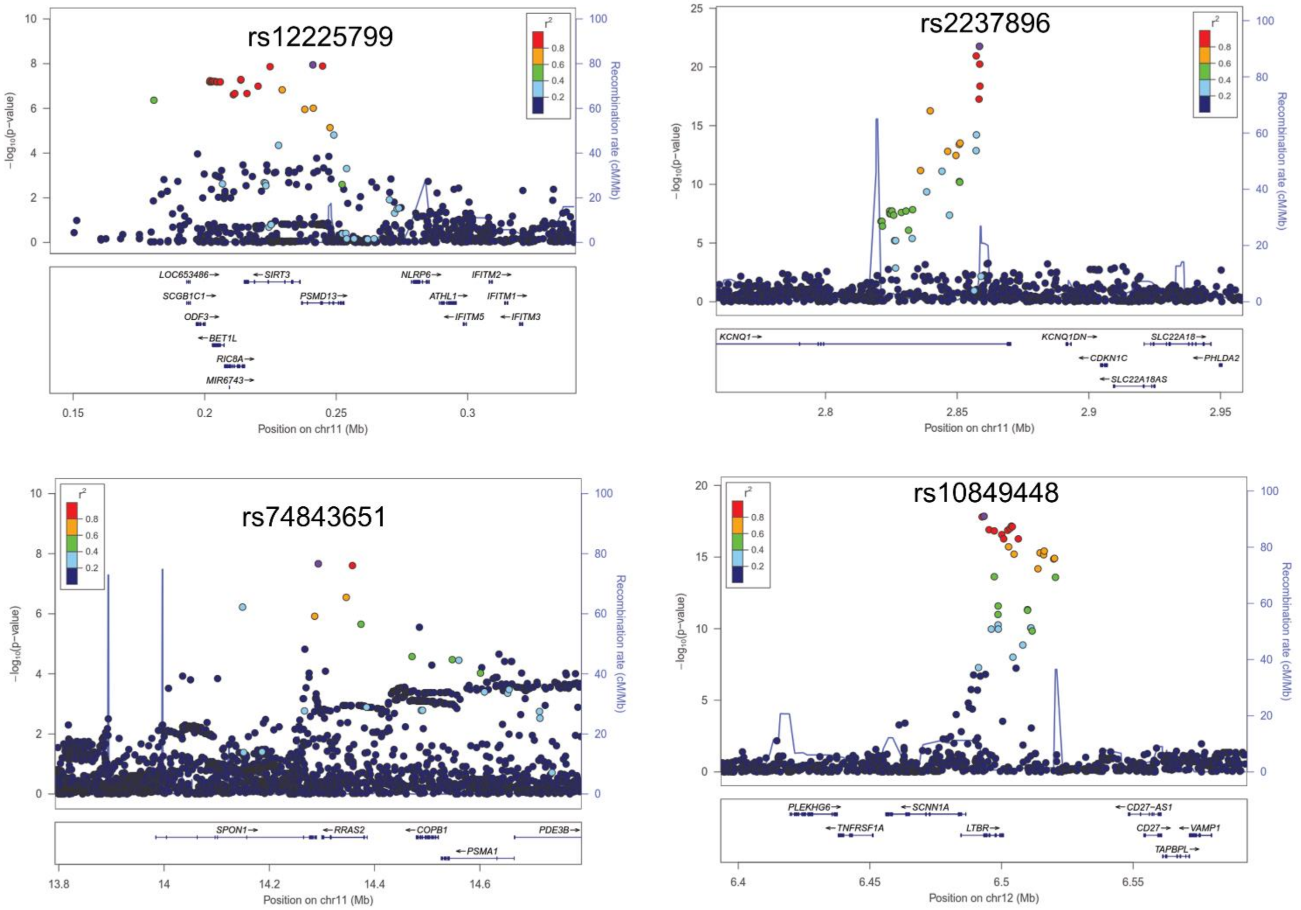
rs12225799
rs2237896
rs74843651
rs10849448
−log₁₀(p−value)
Recombination rate (cM/Mb)
r²
0.8
0.6
0.4
0.2
Position on chr11 (Mb)
Position on chr12 (Mb)
LOC653486→
←SIRT3
SCGB1C1→
PSMD13→
ODF3→
←BET1L
RIC8A→
MIR6743→
NLRP6→
IFITM2→
ATHL1→
IFITM1→
←IFITM5
←IFITM3
KCNQ1→
KCNQ1DN→
SLC22A18→
←CDKN1C
←PHLDA2
←SLC22A18AS
SPON1→
←RRAS2
←COPB1
PDE3B→
←PSMA1
PLEKHG6→
←SCNN1A
←CD27−AS1
←TNFRSF1A
LTBR→
CD27→
←VAMP1
TAPBPL→

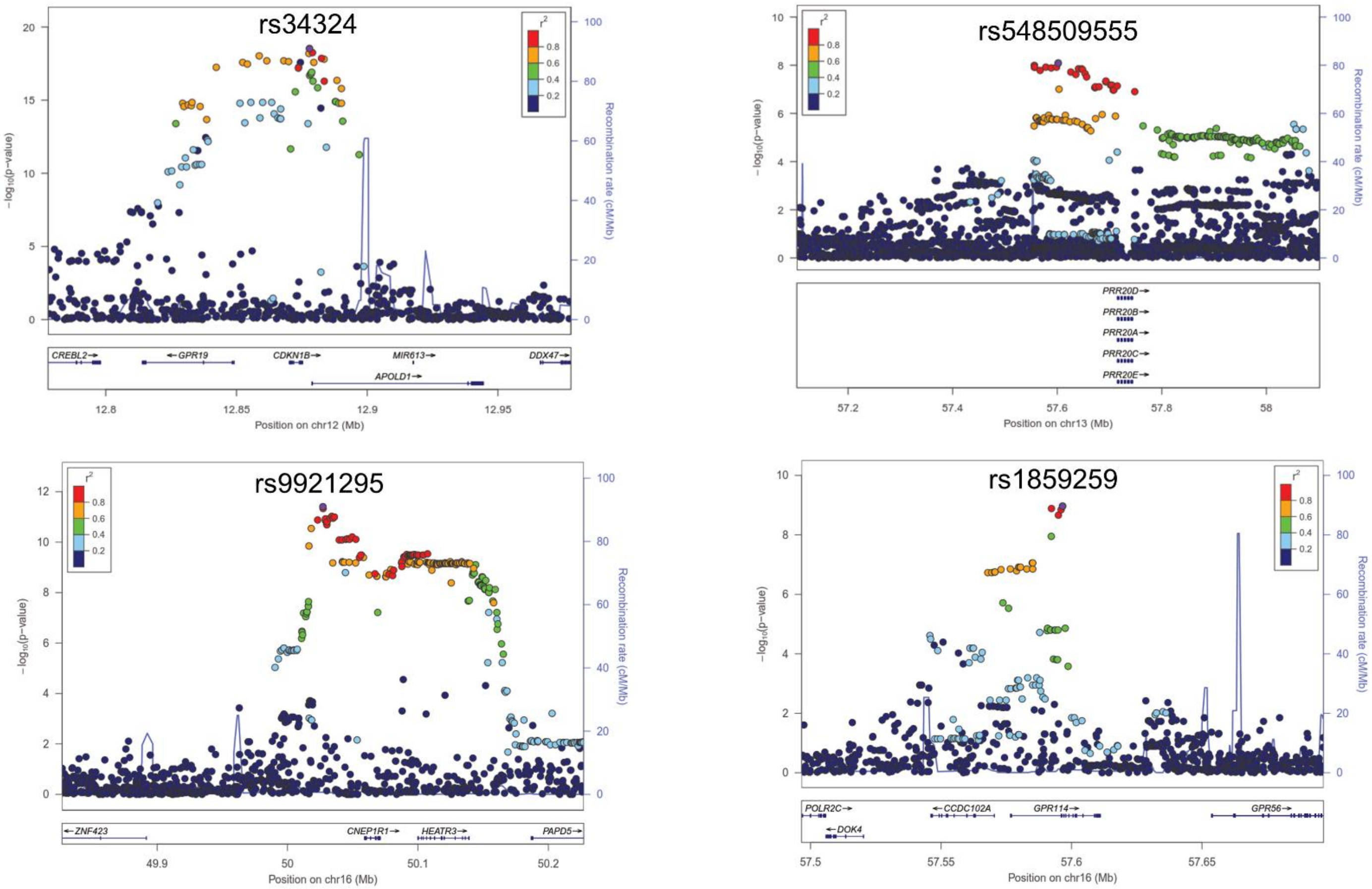
rs34324
−log10(p−value)
Recombination rate (cM/Mb)
r²
CREBL2→
←GPR19
CDKN1B→
MIR613→
DDX47→
APOLD1→
Position on chr12 (Mb)
rs548509555
PRR20D→
PRR20B→
PRR20A→
PRR20C→
PRR20E→
Position on chr13 (Mb)
rs9921295
←ZNF423
CNEP1R1→
HEATR3→
PAPD5→
Position on chr16 (Mb)
rs1859259
POLR2C→
←DOK4
←CCDC102A
GPR114→
GPR56→
Position on chr16 (Mb)

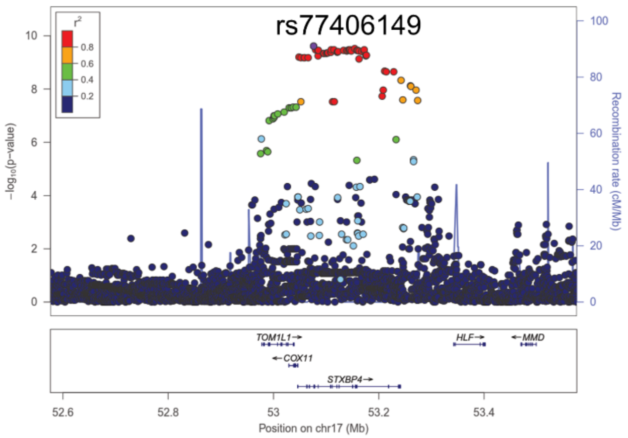
rs77406149
r²
0.8
0.6
0.4
0.2
−log₁₀(p−value)
Recombination rate (cM/Mb)
TOM1L1
COX11
STXBP4
HLF
MMD
52.6
52.8
53
53.2
53.4
Position on chr17 (Mb)

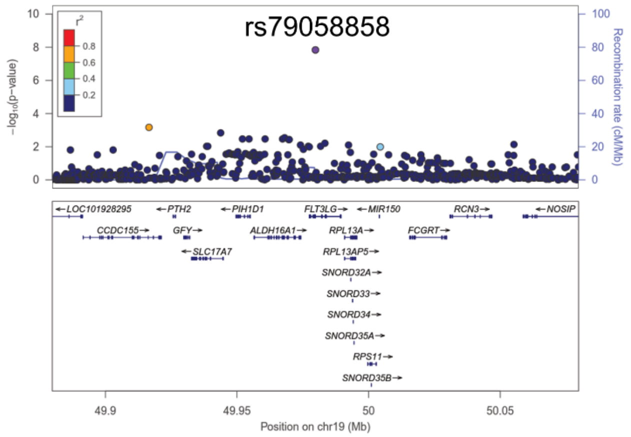
rs79058858
r²
0.8
0.6
0.4
0.2
−log₁₀(p−value)
Recombination rate (cM/Mb)
LOC101928295
PTH2
PIH1D1
FLT3LG
MIR150
RCN3
NOSIP
CCDC155
GFY
ALDH16A1
RPL13A
FCGRT
SLC17A7
RPL13AP5
SNORD32A
SNORD33
SNORD34
SNORD35A
RPS11
SNORD35B
49.9
49.95
50
50.05
Position on chr19 (Mb)

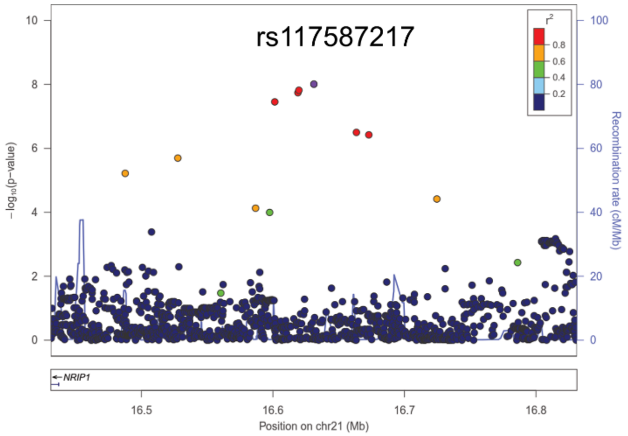
rs117587217
r²
0.8
0.6
0.4
0.2
−log₁₀(p−value)
Recombination rate (cM/Mb)
NRIP1
16.5
16.6
16.7
16.8
Position on chr21 (Mb)

Regional plots of the 31 unreported loci found significant in BBJ are indicated according to chromosomes and chromosomal positions. The most significant variants are plotted in purple and variants in LD with the lead variants are colored according to strength of LD. The range of plots are defined to contain all variants in moderate LD (rsq>0.5) with lead variants.

Supplementary Figure 5. Four loci with two independent signals on mLOY.

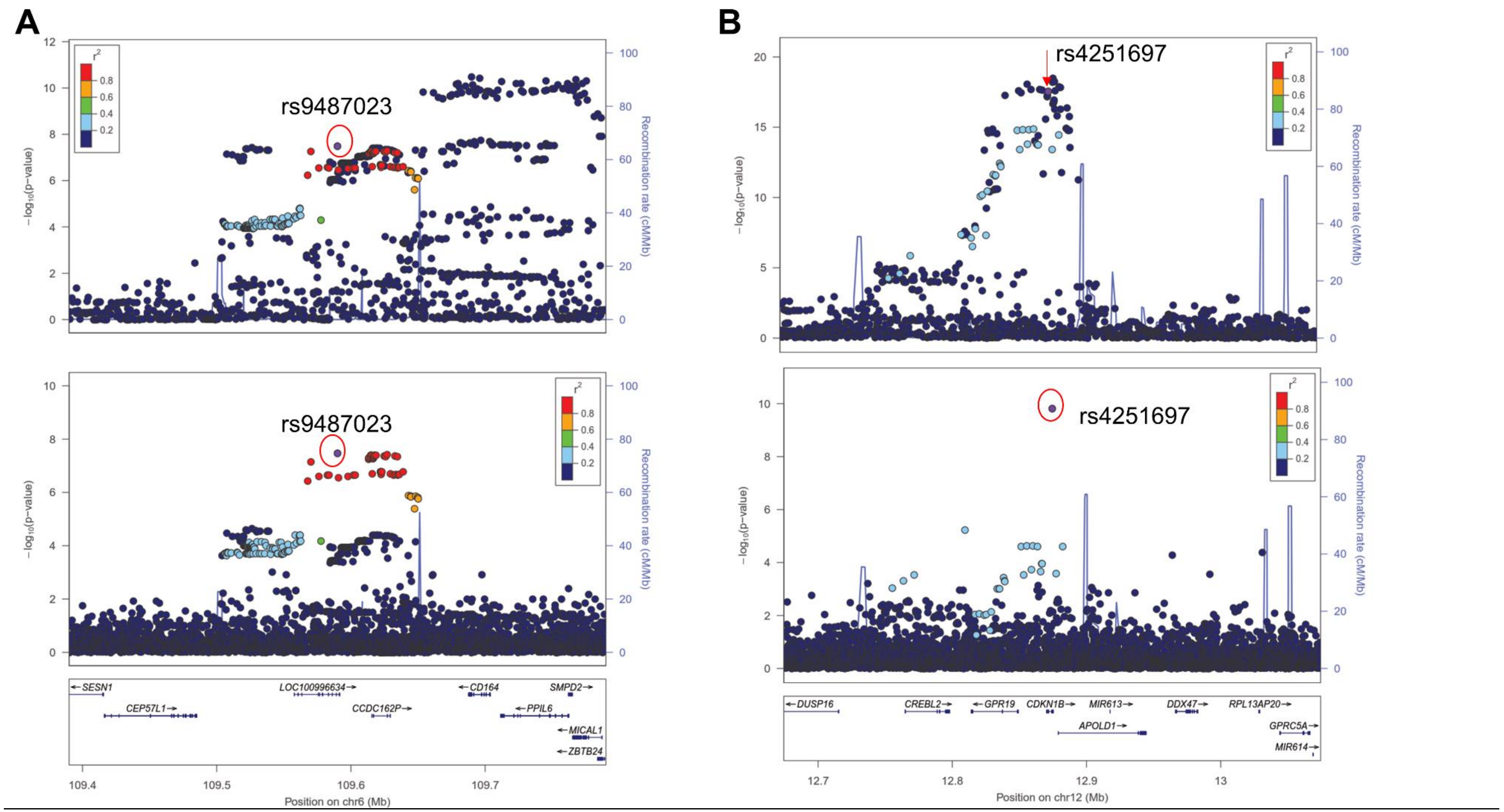

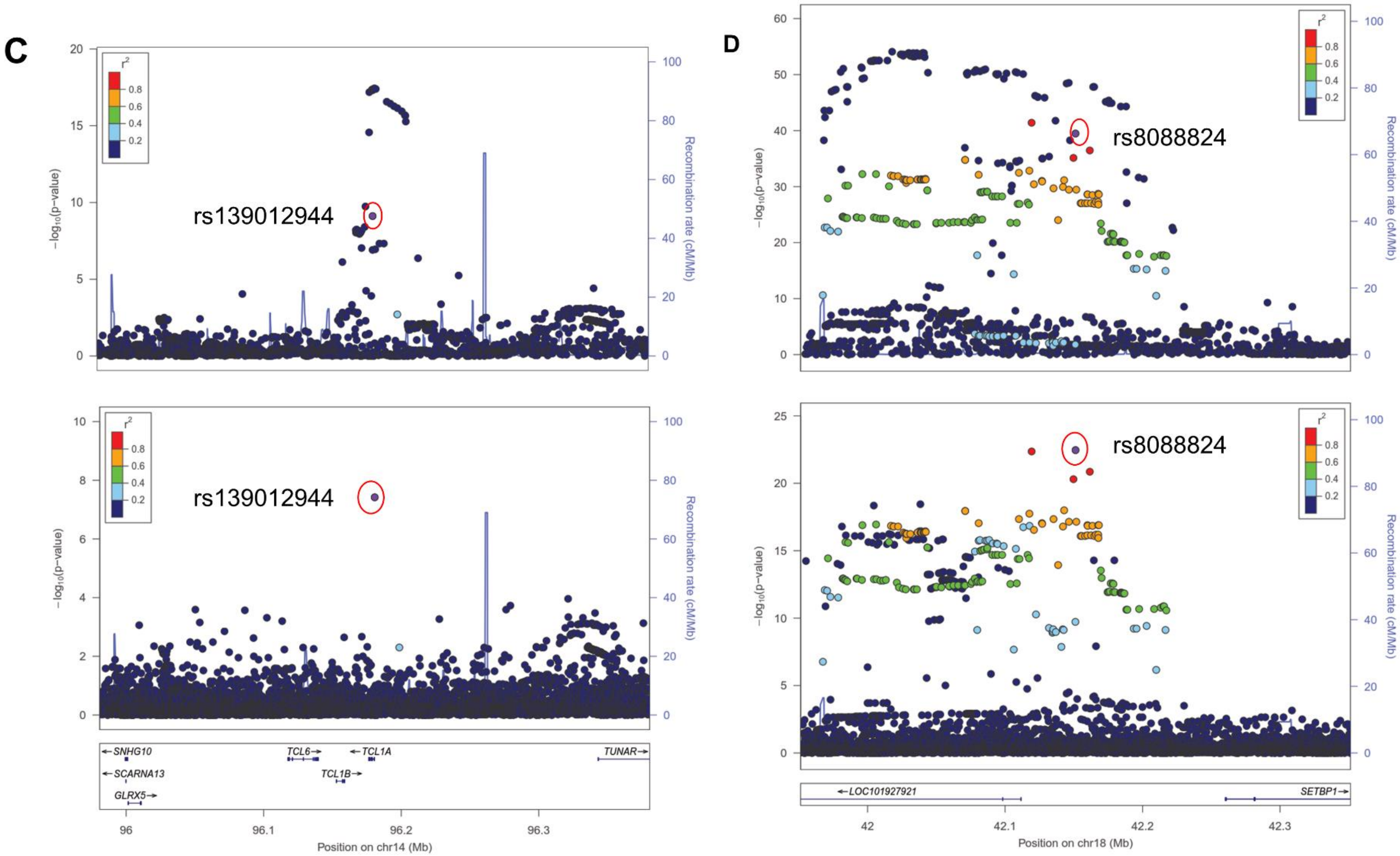

Each of the four regions with two independent signals is illustrated in A-D.

A variant showing the strongest association after conditioning on the top variant in the region is plotted in purple.

Upper and lower plots in each panel indicate results before and after conditioning on the top variant at each locus, respectively.

Supplementary Figure 6. The four loci showing significant associations with mLOY in the UKBB but not in BBJ.

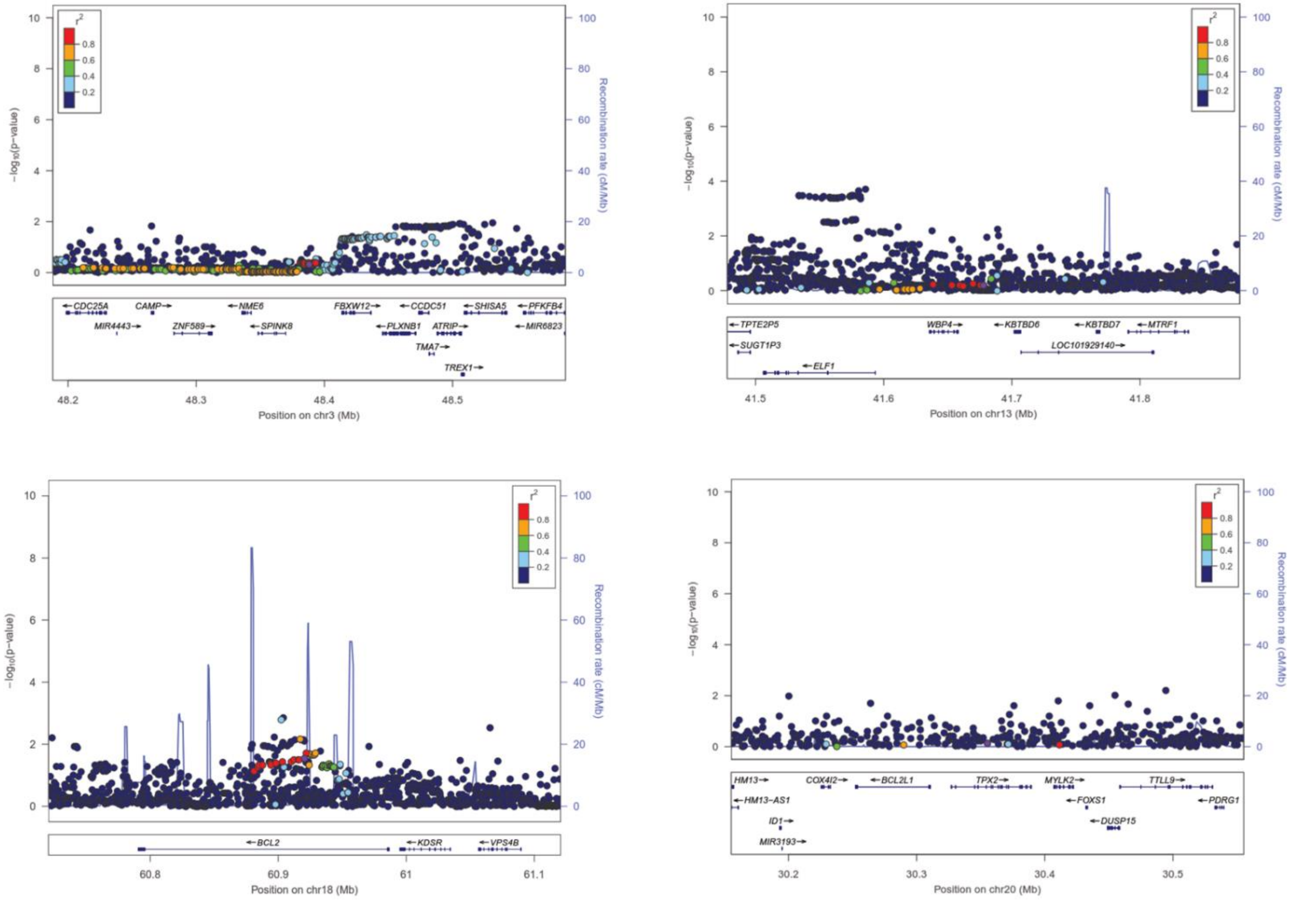

Regional plots of the four loci found significant in UKBB but not in BBJ are indicated. If a top variant in UKBB is present in the BBJ data, the SNP is plotted in purple (we included variants filtered by allele frequency in this plot). Otherwise, we took a variant closest to the UKBB top variant and plotted in purple.

Supplementary Figure 7. The 31 unreported loci in the current study driving the HSC enrichment of mLOY lead variants .

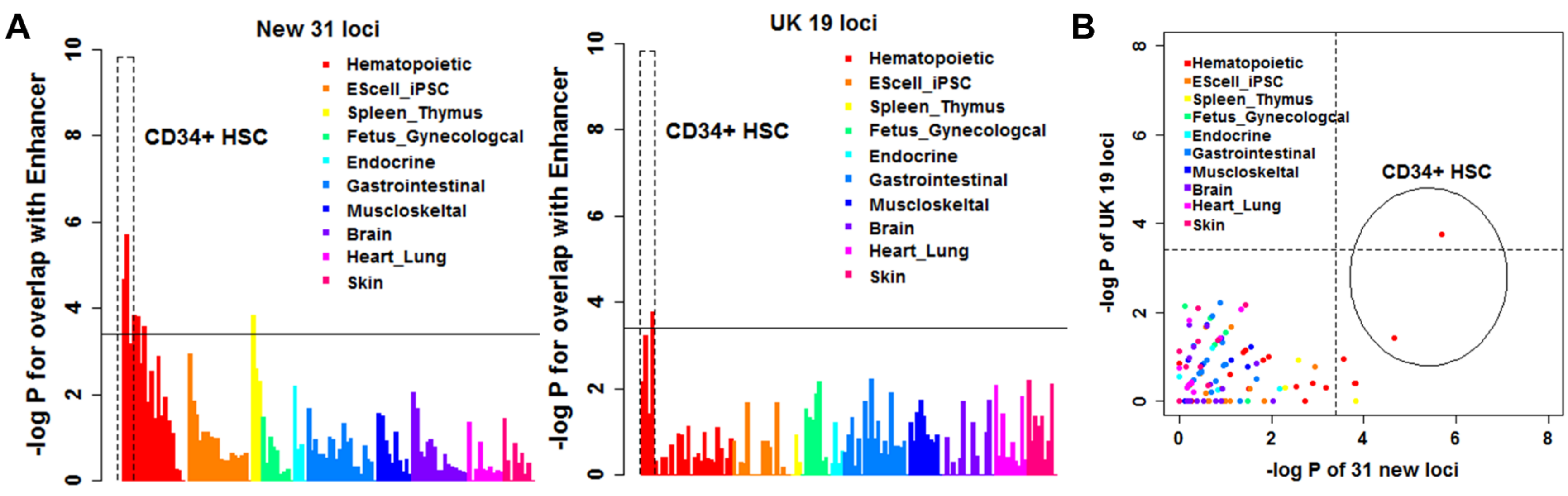

A. Enhancer enrichment was analyzed with the use of Haploreg4.1 software for the new 31 loci (left) and the 19 loci previously reported in the UK population (right). The horizontal solid line indicates the significant level based on Bonferroni's correction (0.05/127).

B. Correlation of p-values for enhancer enrichment in the 127 cells between the 31 loci and the 19 loci is indicated. Broken lines indicate the significant level.

Supplementary Figure 8. Strong heritability enrichment of FLI1 bindings not observed in general.

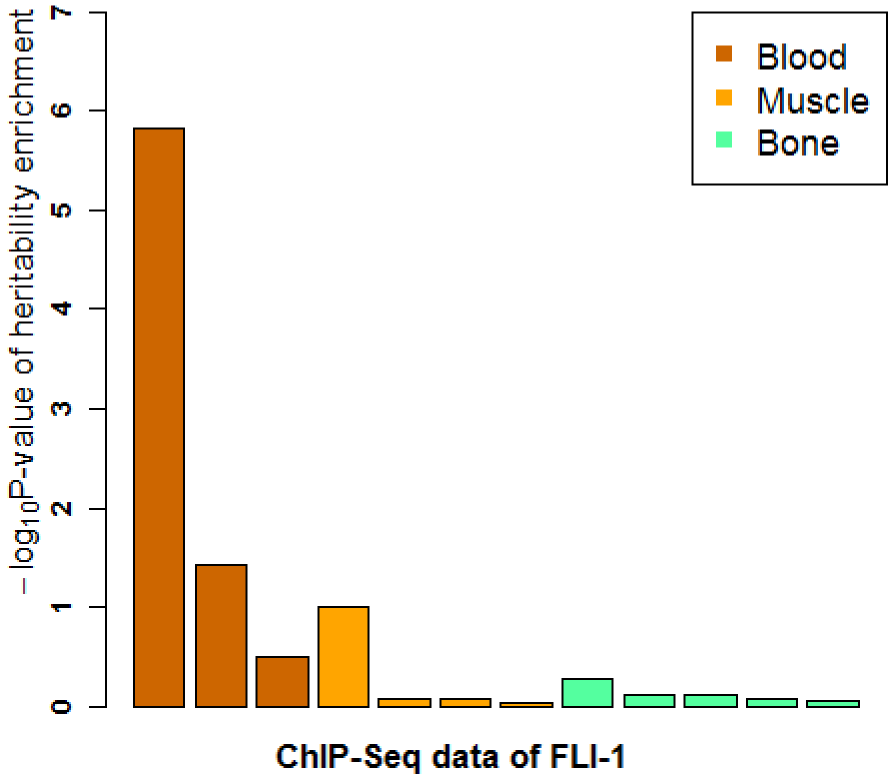

Heritability enrichment of mLOY in ChiP seq data in which FLI1 bindings were analyzed is indicated according to cell types of the data.

Supplementary Figure 9. FLI1 binding in CD34+ cells showing the strongest heritability enrichment in a total of 2,861 ChIP-seq data.

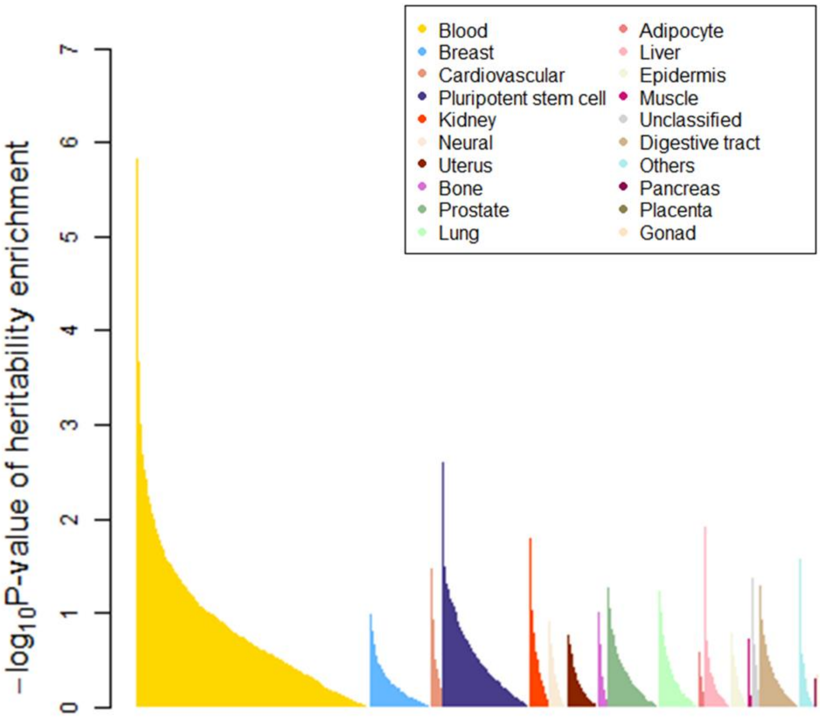

All the results of heritability enrichment of mLOY signals in TF-binding based on 2,861 CHiP seq data are indicated according to p-values and cell types in experiments. Bindings of FLI1 in CD34+ cells (the first bar in the blood cells) showed the strongest heritability enrichment of mLOY.

Supplementary Figure 10. No strong heritability enrichment of mLOY in ChIP seq data using iPS and ES cells.

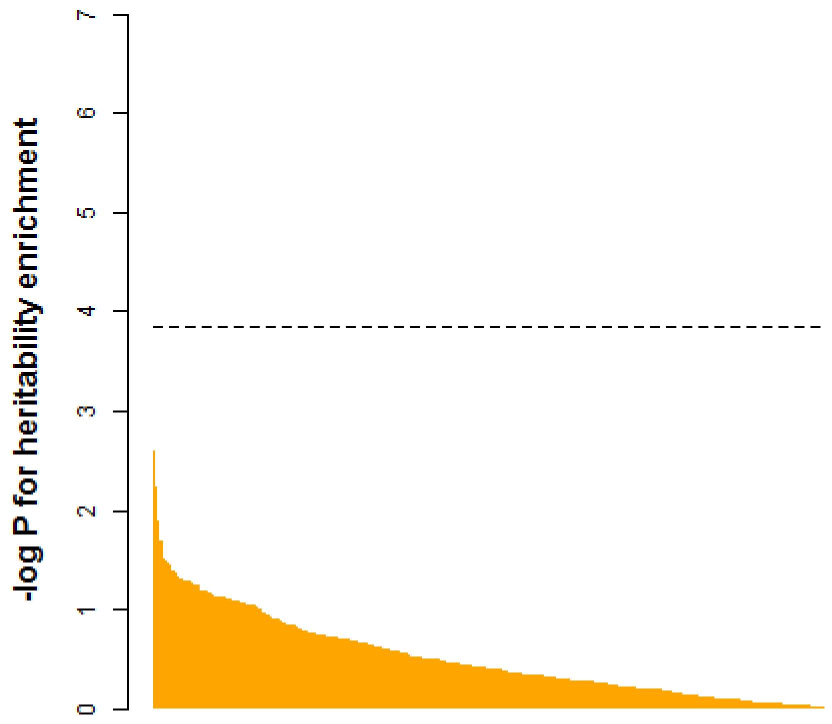

Heritability enrichment of mLOY in TF binding based on ChIP seq data is indicated where transcription factor bindings in iPS or ES cells are analyzed. The results are sorted according to p-values.

Horizontal broken line indicates significant level based on Bonferroni's correction (p=0.00014, p-value for enrichment, 0.05/357)

Supplementary Figure 11. Positive association of mLOY with platelet count, but no or rather an inverse association with RBC count.

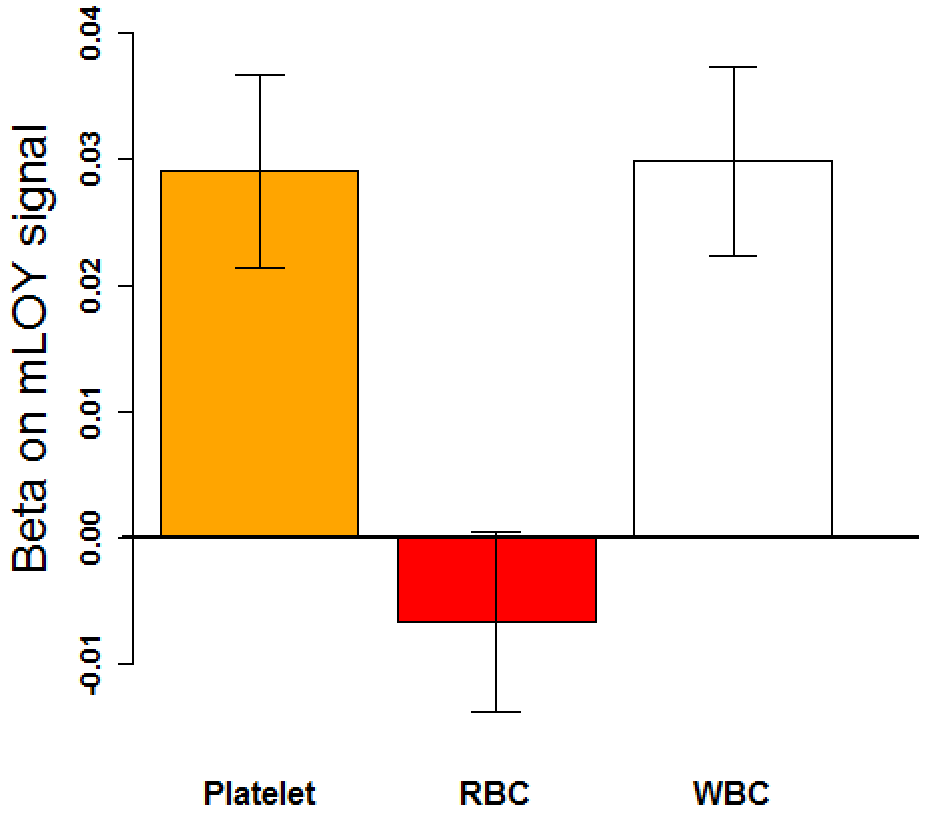

Effect sizes of platelet count, RBC count, and WBC count on mLOY signals conditioning on disease status, data sets, age and smoking are indicated. Effect sizes are estimated in multiple linear regression analyses. Bars indicate 95% confidence interval.

Supplementary Figure 12. A causal effect of mLOY on AST suggested by Mendelian randomization.

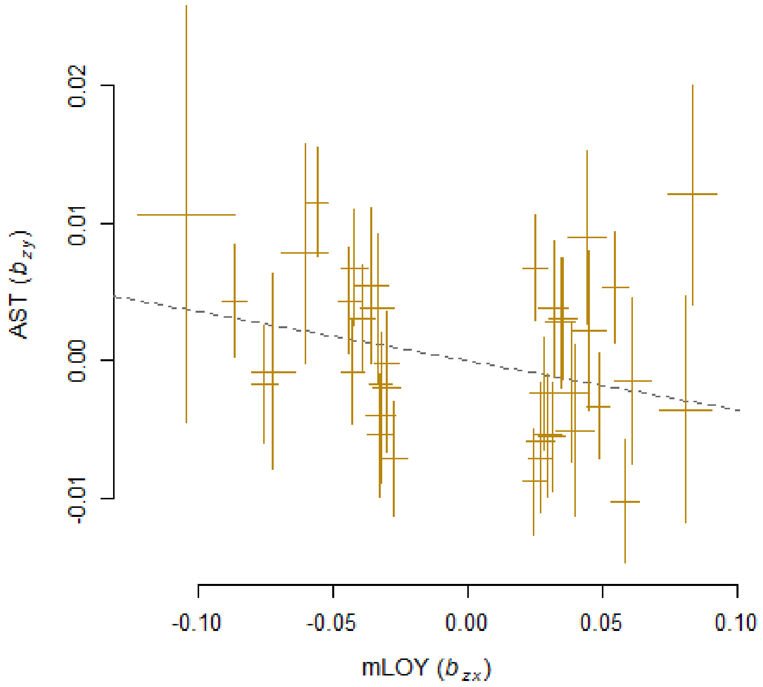

Variants significantly associated with mLOY are extracted and filtered based on strict LD filter (rsq>0.05) and HEIDI-outliers. Effect sizes on mLOY (mLRR-Y) and levels of AST in the remaining variants are plotted. Error bars indicate standard errors of beta.

Supplementary Figure 13. Associations between overall mortality and top mLOY signals.

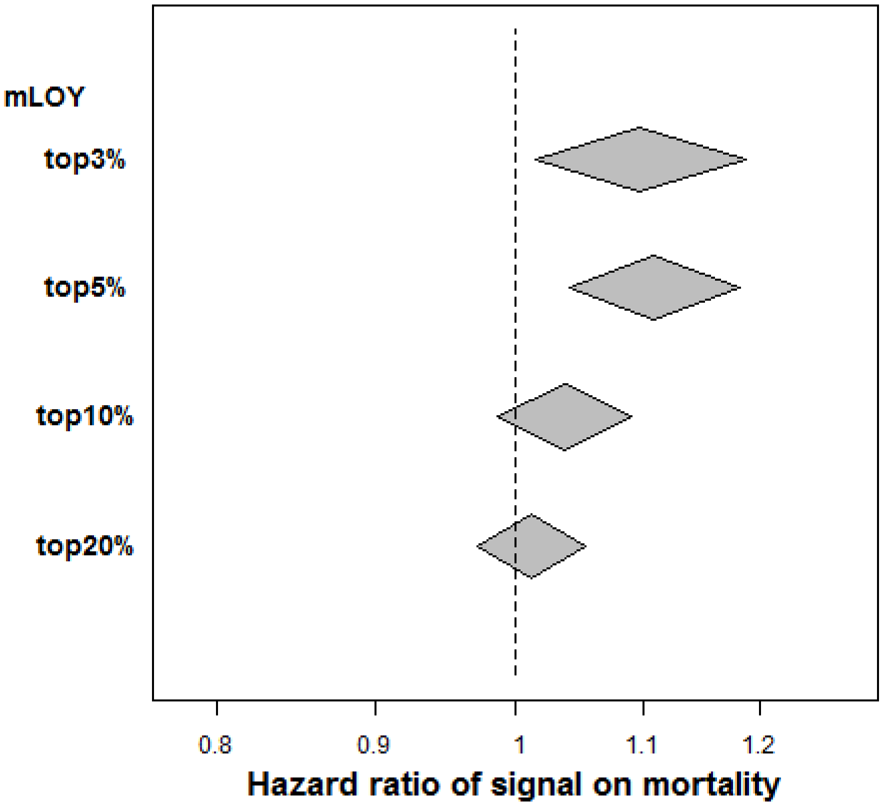

Hazard ratio of subjects who had top mLOY signals on overall mortality is indicated. Width of diamonds indicates 95% confidence interval of hazard ratio. Disease status, data sets, age and smoking are used for covariates.

A vertical broken line indicates hazard ratio of 1.0.

Supplementary Figure 14. Associations between death from lung cancer and subjects having top mLOY signal.

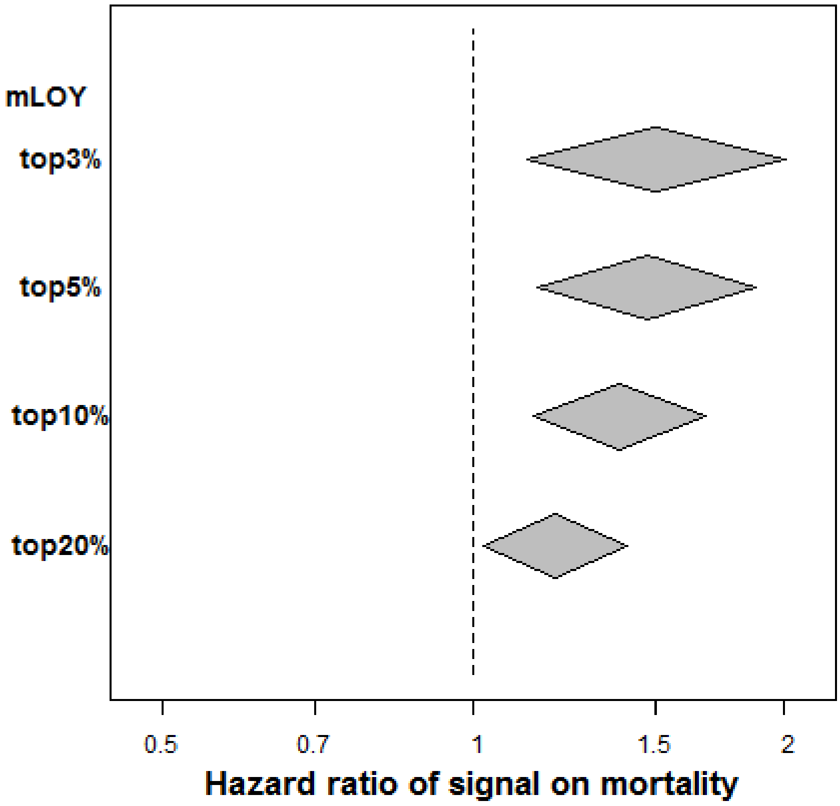

Hazard ratio of subjects who had top mLOY signals on lung cancer mortality is indicated. Disease status, data sets, age and smoking are used as covariates. Width of diamonds indicates 95% confidence interval of hazard ratio. A vertical broken line indicates hazard ratio of 1.0.

Supplementary Figure 15. No significant genetic correlations between mLOY and cancer susceptibility.

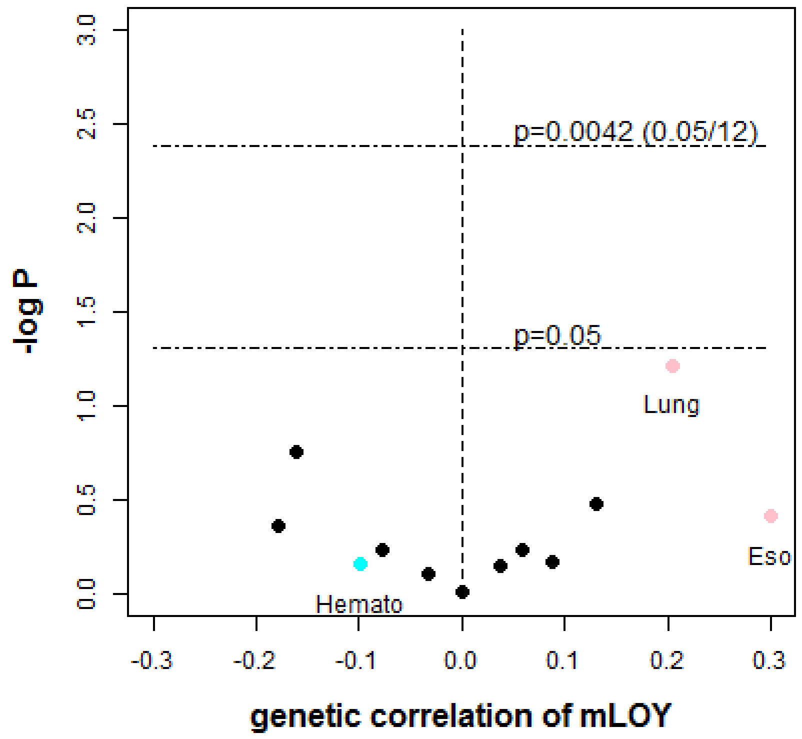

Genetic correlations of mLOY are plotted in a total of 12 cancers/malignancies.
Lung cancer, esophagus cancer and hematopoietic malignancies are shown in different colors from the other malignancies since these showed the strongest association, the strongest effect size and their involvements with mLOY were previously reported, respectively.

Hemato: Hematopoietic malignancy
Lung: Lung cancer
Eso: Esophagus cancer

Supplementary Table 1. Information of subjects analyzed in the three data sets in the BBJ.

	Set 1	Set 2	Set 3
Array	OmniExpressExome v1.2	OmniExpressExome v1.0	OmniExpress v1.0 and HumanExome v1.0, 1.1
N (QCed)	58,985	18,336	18,059
Age (mean±SD)	61.7±14.0	67.7±10.3	62.6±15.1
Smoker ratio*	0.746	0.742	0.759
Hematopoietic malignancy	0.06%	0.04%	3.00%
Non-hematopoietic malignancy	8.90%	2.50%	58.50%

*since detailed information of smoking (duration and quantity) was available for a limited number of subjects (37% of smokers), we define two categories of smoking, smoker and non-smoker (never-smokers).

Deviation of distribution of diseases is explained in Supplementary Note 8.

Supplementary Table 2. Effect sizes and p-values of the top markers in the 46 significant loci in the three data sets.

SNP	BP	Gene	A0	A1	Set1 A1Beta	Set1 P	Set2 A1Beta	Set2 P	Set3 A1Beta	Set3 P	Direction of effect in 3 batches	I2
Unreported												
rs34468831	1:3097312	*PRDM16*	GA	G	-0.037	$2.2x10^{-10}$	-0.035	$1.3x10^{-3}$	-0.0097	$3.7x10^{-1}$	---	66.1
rs527504	1:33392427	*TMEM54;RNF19B*	G	A	-0.033	$3.8x10^{-6}$	-0.031	$1.7x10^{-2}$	-0.031	$1.0x10^{-2}$	---	0
rs17049722	2:58976863	*LINC01122*	C	T	0.038	$2.4x10^{-7}$	0.032	$2.1x10^{-2}$	0.045	$2.6x10^{-4}$	+++	0
2:136879065	2:136879065	*CXCR4*	G	ALU	-0.033	$4.6x10^{-7}$	-0.036	$9.0x10^{-4}$	-0.038	$3.0x10^{-4}$	---	0
rs34778241	3:71771215	*EIF4E3*	T	TG	-0.026	$9.1x10^{-5}$	-0.039	$1.2x10^{-3}$	-0.033	$2.9x10^{-3}$	---	0
rs2811487	3:128331879	*LINC01565;RPN1*	G	A	0.026	$3.2x10^{-6}$	0.037	$2.0x10^{-4}$	0.02	$3.2x10^{-2}$	+++	0
rs871134	4:7044380	*CCDC96*	C	T	-0.031	$4.5x10^{-9}$	-0.037	$3.3x10^{-4}$	-0.025	$8.3x10^{-3}$	---	0
rs2853677	5:1287194	*TERT*	G	A	0.03	$8.2x10^{-7}$	0.025	$1.8x10^{-2}$	0.03	$2.5x10^{-3}$	+++	0
rs10948011	6:42024285	*TAF8*	G	A	0.038	$2.3x10^{-9}$	0.031	$8.7x10^{-3}$	0.032	$3.0x10^{-3}$	+++	0
rs35355140	7:27204732	*HOXA9*	C	A	0.045	$3.3x10^{-8}$	0.046	$6.7x10^{-4}$	0.039	$2.7x10^{-3}$	+++	0
rs11769630	7:50257703	*C7orf72;IKZF1*	T	A	-0.056	$7.2x10^{-12}$	-0.073	$3.5x10^{-7}$	-0.037	$7.3x10^{-3}$	---	38.1

rs59543286	7:135351310	*C7orf73*	A	C	0.061	9.6x10^{-21}	0.064	7.6x10^{-8}	0.046	3.0x10^{-5}	+++	0
rs55727837	7:149428602	*KRBA1*	G	T	0.074	9.8x10^{-17}	0.035	2.9x10^{-2}	0.051	4.4x10^{-4}	+++	61.7
rs12668837	7:158500805	*NCAPG2;ESYT2*	C	T	-0.024	1.2x10^{-5}	-0.026	6.2x10^{-3}	-0.032	8.2x10^{-4}	---	0
rs189309686	8:59509355	*NSMAF*	C	T	0.024	8.5x10^{-5}	0.048	1.8x10^{-5}	0.015	1.6x10^{-1}	+++	60.8
rs10692222	8:130597362	*CCDC26*	C	CATT	0.021	1.5x10^{-4}	0.025	1.0x10^{-2}	0.03	1.4x10^{-3}	+++	0
rs2804301	9:603916	*KANK1*	G	A	-0.03	1.9x10^{-6}	-0.04	4.9x10^{-4}	-0.0004	9.7x10^{-1}	---	73.5
rs9299129	9:109638167	*ZNF462*	A	G	0.035	7.2x10^{-8}	0.028	1.7x10^{-2}	0.015	2.0x10^{-1}	+++	21.9
rs138423884	9:129855937	*ANGPTL2; RALGPS1*	A	G	0.093	7.9x10^{-11}	0.048	9.6x10^{-2}	0.062	1.5x10^{-2}	+++	26.8
rs2646425	10:8470387	*LINC00708; LOC105376398*	C	T	0.021	8.4x10^{-4}	0.039	2.8x10^{-4}	0.034	9.4x10^{-4}	+++	29.5
rs12225799	11:241124	*PSMD13*	C	G	-0.048	7.7x10^{-9}	-0.012	4.3x10^{-1}	-0.022	1.2x10^{-1}	---	65.2
rs2237896	11:2858440	*KCNQ1*	G	A	0.046	3.5x10^{-16}	0.035	6.8x10^{-4}	0.036	1.2x10^{-4}	+++	0
rs74843651	11:14292987	*SPON1;RRAS2*	G	T	0.047	3.5x10^{-4}	0.069	2.5x10^{-3}	0.056	7.9x10^{-3}	+++	0
rs10849448	12:6493351	*LTBR*	A	G	-0.046	9.5x10^{-11}	-0.054	4.9x10^{-5}	-0.051	3.7x10^{-5}	---	0

rs34324	12:12877926	*APOLD1*	C	A	-0.041	3.6x10^{-13}	-0.028	5.0x10^{-3}	-0.042	1.6x10^{-5}	---	0
rs548509555	13:57601900	*MIR5007;PRR20B*	T	TA	0.031	2.0x10^{-5}	0.041	8.0x10^{-4}	0.028	1.7x10^{-2}	+++	0
rs9921295	16:50027130	*ZNF423;CNEP1R1*	T	G	0.032	5.5x10^{-8}	0.027	1.3x10^{-2}	0.031	1.0x10^{-3}	+++	0
rs1859259	16:57596552	*ADGRG5*	C	T	-0.029	5.0x10^{-7}	-0.018	5.2x10^{-2}	-0.02	3.3x10^{-2}	---	0
rs77406149	17:53076247	*STXBP4*	A	G	0.032	4.8x10^{-6}	0.038	2.9x10^{-3}	0.039	8.3x10^{-4}	+++	0
rs79058858	19:49979789	*FLT3LG*	C	T	0.16	4.8x10^{-5}	0.23	1.4x10^{-3}	0.16	1.2x10^{-2}	+++	0
rs117587217	21:16631171	*NRIP1;USP25*	T	C	-0.12	3.2x10^{-7}	-0.13	3.1x10^{-3}	-0.026	5.1x10^{-1}	---	54.8
known												
rs2842873	1:156204653	*PMF1;PMF1-BGLAP*	C	T	-0.055	1.4x10^{-22}	-0.056	2.4x10^{-8}	-0.053	2.4x10^{-8}	---	0
rs4683900	3:101134696	*SENP7*	C	T	0.035	8.3x10^{-9}	0.069	1.1x10^{-9}	0.041	9.2x10^{-5}	+++	70.9
rs4681200	3:150002138	*LINC01214*	C	G	0.077	4.4x10^{-13}	0.067	4.2x10^{-4}	0.056	9.6x10^{-4}	+++	0
rs56116444	5:111061847	*STARD4-AS1*	T	G	-0.081	1.0x10^{-43}	-0.088	3.8x10^{-17}	-0.099	2.0x10^{-23}	---	13.3
rs11251	6:109689907	*CD164*	G	T	0.03	5.0x10^{-8}	0.035	4.9x10^{-4}	0.019	4.7x10^{-2}	+++	0
rs4709819	6:164463355	*LOC102724152;MEAT6*	G	A	0.049	4.0x10^{-19}	0.056	4.0x10^{-8}	0.039	2.5x10^{-5}	+++	0

rs4721217	7:1973579	*MAD1L1*	C	T	-0.042	4.4×10^{-14}	-0.05	1.0×10^{-6}	-0.033	4.7×10^{-4}	---	0
rs2979469	8:30285091	*RBPMS*	G	C	-0.034	6.7×10^{-5}	-0.042	4.1×10^{-3}	-0.035	1.1×10^{-2}	---	0
rs227079	11:108248686	*C11orf65*	C	A	-0.044	2.7×10^{-15}	-0.043	6.4×10^{-6}	-0.046	5.9×10^{-7}	---	0
rs728739	14:96182062	*TCL1A;TUNAR*	A	G	0.081	1.6×10^{-11}	0.084	7.5×10^{-5}	0.076	1.4×10^{-4}	+++	0
rs72698721	14:101181189	*LINC00523; DLK1*	G	A	0.055	3.7×10^{-21}	0.061	6.1×10^{-9}	0.047	1.4×10^{-6}	+++	0
rs77874075	16:81066339	*CENPN*	T	G	-0.031	1.9×10^{-6}	-0.04	1.2×10^{-3}	-0.018	1.0×10^{-1}	---	0
rs201753350	17:7579705	*TP53*	C	T	-0.15	1.4×10^{-5}	-0.17	1.7×10^{-2}	-0.13	2.8×10^{-2}	---	0
rs78997619	17:47787161	*FAM117A*	C	T	0.095	1.3×10^{-15}	0.062	5.9×10^{-3}	0.055	5.4×10^{-3}	+++	50.5
rs80277818	18:42017901	*LINC01478*	A	G	-0.079	1.4×10^{-36}	-0.058	3.9×10^{-7}	-0.081	5.4×10^{-15}	---	35.5

Chr:chromosome, BP:base pair position in NCBI build 37, Gene: italicized gene name

Supplementary Table 3. The four variants showing significant associations in the UKBB but not in the BBJ.

SNPID	Position	Gene	Allele	UK_Af	UK_P	BBJ_Af	BBJ_P	Reason of NA
rs17758695	18q21.33	*BCL2*	C/T	0.97	$1.3x10^{-33}$	NA	NA	Monomorphic in Japanese but contained in the Ref panel (resulting in low rsq (0.13))
rs60084722	20q11.21	*TPX2,BCL2L1,HM13*	CT/C	0.79	$1.6x10^{-17}$	NA	NA	Monomorphic in Japanese and not in the Ref panel
rs10687116	13q14.11	*WBP4*	AGATG/A	0.8	$8.8x10^{-10}$	NA	NA	Monomorphic in Japanese and not in the Ref panel
rs115854006	3p21.31	*TREX1,PLXNB1*	C/T	0.96	$4.5x10^{-8}$	0.0016	NA	filtered by low af (0.001)

Gene: italicized gene name

Supplementary Table 4. Common genetic architecture of mLOY between BBJ and UKBB.

							BBJ			UKBB			
Category	SNP	Chr	Basepair position	Gene	Allele	Test allele	Test allele freq	Test allele beta	P	Test allele freq	Test allele beta	P	Dir
Unreported	rs34468831	1	3097312	*PRDM16*	GA/G	G	0.37	-0.032	3×10^{-12}	0.09	0.012	6×10^{-8}	1
Unreported	rs527504	1	33392427	*TMEM54;RNF19B*	G/A	A	0.19	-0.032	4.1×10^{-9}	0.2	0.006	0.00011	1
Unreported	rs17049722	2	58976863	*LINC01122*	C/T	C	0.83	0.038	4.4×10^{-12}	0.86	0.009	8.4×10^{-8}	1
Unreported	2:136879065	2	136879065	*CXCR4*	G/ALU	ALU	0.32	-0.035	9.3×10^{-13}				
Unreported	rs34778241	3	71771215	*EIF4E3*	T/TG	TG	0.71	-0.03	2.1×10^{-9}	0.62	0.013	1.2×10^{-24}	1
Unreported	rs2811487	3	128331879	*LINC01565;RPN1*	G/A	G	0.64	0.027	6.8×10^{-10}	0.77	0.01	3.4×10^{-12}	1
Unreported	rs871134	4	7044380	*CCDC96*	C/T	T	0.49	-0.032	1.4×10^{-14}	0.57	0.01	1.4×10^{-16}	1
Unreported	rs2853677	5	1287194	*TERT*	G/A	G	0.3	0.027	2.1×10^{-9}	0.42	0.005	8.1×10^{-6}	1
Unreported	rs10948011	6	42024285	*TAF8*	G/A	G	0.77	0.035	4×10^{-12}	0.87	0.022	9.4×10^{-36}	1

Unreported	rs35355140	7	27204732	*HOXA9*	C/A	C	0.86	0.045	2.3×10^{-13}	0.93	0.023	8.8×10^{-25}	1
Unreported	rs11769630	7	50257703	*C7orf72;IKZF1*	T/A	A	0.13	-0.057	3.7×10^{-19}	0.07	0.021	3.9×10^{-21}	1
Unreported	rs59543286	7	135351310	*C7orf73*	A/C	A	0.77	0.059	8×10^{-32}	0.84	0.021	2.3×10^{-38}	1
Unreported	rs55727837	7	149428602	*KRBA1*	G/T	G	0.89	0.061	9.7×10^{-20}	0.89	0.022	1.3×10^{-34}	1
Unreported	rs12668837	7	158500805	*NCAPG2;ESYT2*	C/T	T	0.46	-0.026	1.8×10^{-9}	0.08	0.011	1.1×10^{-6}	1
Unreported	rs189309686	8	59509355	*NSMAF*	C/T	C	0.75	0.027	4.4×10^{-8}	0.999	-0.005	0.82	0
Unreported	rs10692222	8	130597362	*CCDC26*	C/CATT	C	0.57	0.025	6×10^{-9}	0.73	0.005	4.9×10^{-5}	1
Unreported	rs2804301	9	603916	*KANK1*	G/A	A	0.24	-0.027	4.4×10^{-8}	0.53	0.006	1.5×10^{-6}	1
Unreported	rs9299129	9	109638167	*ZNF462*	A/G	A	0.77	0.03	2.2×10^{-9}	0.9	0.007	0.00019	1
Unreported	rs138423884	9	129855937	*ANGPTL2;RALGPS1*	A/G	A	0.962	0.079	1.3×10^{-12}				
Unreported	rs2646425	10	8470387	*LINC00708;LOC105376398*	C/T	C	0.69	0.028	4.3×10^{-10}	0.54	0.004	0.0014	1

Unreported	rs12225799	11	241124	*PSMD13*	C/G	G	0.13	-0.036	1.1x10^{-8}	0.04	0.005	0.067	1
Unreported	rs2237896	11	2858440	*KCNQ1*	G/A	G	0.6	0.043	1.7x10^{-22}	0.96	0.017	7.2 x10^{-9}	1
Unreported	rs74843651	11	14292987	*SPON1;RRAS2*	G/T	G	0.948	0.055	2.2x10^{-8}				
Unreported	rs10849448	12	6493351	*LTBR*	A/G	G	0.81	-0.05	1.5x10^{-18}	0.75	0.013	3.7 x10^{-22}	1
Unreported	rs34324	12	12877926	*APOLD1*	C/A	A	0.43	-0.039	3.1x10^{-19}	0.4	0.009	3.4 x10^{-15}	1
Unreported	rs548509555	13	57601900	*MIR5007;PRR20B*	T/TA	T	0.8	0.032	8.1x10^{-9}	0.9	0	0.93	0
Unreported	rs9921295	16	50027130	*ZNF423;CNEP1R1*	T/G	T	0.67	0.031	4x10^{-12}	0.92	0.01	7.8 x10^{-7}	1
Unreported	rs1859259	16	57596552	*GPR114*	C/T	T	0.58	-0.025	1.1x10^{-9}	0.3	0.008	9.5 x10^{-12}	1
Unreported	rs77406149	17	53076247	*STXBP4*	A/G	A	0.8	0.035	2.5x10^{-10}	0.98	-0.001	0.73	0
Unreported	rs79058858	19	49979789	*FLT3LG*	C/T	C	0.9948	0.16	1.4x10^{-8}				
Unreported	rs117587217	21	16631171	*NRIP1;USP25*	T/C	C	0.016	-0.1	9.7x10^{-9}				

Known	rs2842873	1	156204653	*PMF1;PMF1-BGLAP*	C/T	T	0.37	-0.056	1.5×10^{-36}	0.37	0.024	3.2×10^{-88}	1
Known	rs4683900	3	101134696	*SENP7*	C/T	C	0.27	0.042	7.9×10^{-19}	0.38	0.016	5.6×10^{-44}	1
Known	rs4681200	3	150002138	*LINC01214*	C/G	C	0.08	0.072	3.8×10^{-19}	0.21	0.029	4.6×10^{-90}	1
Known	rs56116444	5	111061847	*STARD4-AS1*	T/G	G	0.31	-0.086	9.2×10^{-81}	0.07	0.027	9.3×10^{-36}	1
Known	rs11251	6	109689907	*CD164*	G/T	G	0.46	0.028	3.4×10^{-11}	0.61	0.013	1.7×10^{-30}	1
Known	rs4709819	6	164463355	*LOC102724152;MEAT6*	G/A	G	0.59	0.049	1.3×10^{-30}	0.59	0.023	3.2×10^{-83}	1
Known	rs4721217	7	1973579	*MAD1L1*	C/T	T	0.42	-0.043	6.8×10^{-23}	0.4	0.019	2.5×10^{-55}	1
Known	rs2979469	8	30285091	*RBPMS*	G/C	C	0.88	-0.035	4.9×10^{-8}	0.74	0.015	2.8×10^{-30}	1
Known	rs227079	11	108248686	*C11orf65*	C/A	A	0.48	-0.044	5.9×10^{-26}	0.55	0.014	8.9×10^{-31}	1
Known	rs728739	14	96182062	*TCL1A;TUNAR*	A/G	A	0.945	0.081	3.8×10^{-18}	0.84	0.035	2×10^{-106}	1
Known	rs72698721	14	101181189	*LINC00523;DLK1*	G/A	G	0.68	0.055	4.2×10^{-33}	0.87	0.026	1.4×10^{-52}	1
Known	rs77874075	16	81066339	*CENPN*	T/G	G	0.22	-0.03	4.6×10^{-9}	0.09	0.021	4.9×10^{-25}	1
Known	rs201753350	17	7579705	*TP53*	C/T	T	0.0062	-0.15	1×10^{-8}				
Known	rs78997619	17	47787161	*FAM117A*	C/T	C	0.94	0.083	2.5×10^{-21}	0.96	0.037	1.2×10^{-38}	1
Known	rs80277818	18	42017901	*LINC01478*	A/G	G	0.26	-0.075	7.8×10^{-55}	0.13	0.033	3×10^{-80}	1

Gene:italicized gene name,Dir:common direction of effect in tested allele

Supplementary Table 5. Cells whose enhancers significantly overlapped with the top 46 variants by Haploreg.

Cell	P value
Primary_hematopoietic_stem_cells_G-CSF-mobilized_Female	2.6×10^{-10}
Primary_hematopoietic_stem_cells_short_term_culture	7.1×10^{-7}
Primary_hematopoietic_stem_cells_G-CSF-mobilized_Male	1.0×10^{-6}
Primary_hematopoietic_stem_cells	6.0×10^{-6}
Primary_T_helper_cells_PMA-I_stimulated	0.00019
Primary_mononuclear_cells_from peripheral blood	0.0002
Primary_T_helper_17_cells_PMA-I_stimulated	0.00021
Primary_B_cells_from_cord_blood	0.00021
Monocytes-CD14+_RO01746_Primary_Cells	0.00036
ES-UCSF4_Cells	0.00038

Supplementary Table 6. LDSC results with 53 basic annotations for mLOY.

	Prop.SNPs	Heritability enrichment	P value
SuperEnhancer_HniszL2_0	0.167642	3.206788	4.3×10^{-9}
H3K27ac_HniszL2_0	0.388626	2.54295	1.7×10^{-8}
TSS_Hoffman.extend.500L2_0	0.034492	13.15701	3.1×10^{-6}
H3K27ac_PGC2.extend.500L2_0	0.335339	2.600229	0.00012
H3K4me1_TrynkaL2_0	0.420643	2.699539	0.00014
Promoter_UCSC.extend.500L2_0	0.038113	6.245748	0.00037
Enhancer_Hoffman.extend.500L2_0	0.08923	5.279289	0.00043
Intron_UCSC.extend.500L2_0	0.396159	1.505862	0.00056

Results above significant level (0.05/53) are indicated. Prop. SNPs: proportion of SNPs

Supplementary Table 7. Cells and histone marks showing significant heritability enrichment in mLOY.

Cell	Histone Mark	P value
Mobilized_CD34_primary	H3K4me1	6.59×10^{-10}
Mobilized_CD34_primary	H3K4me3	2.72×10^{-8}
CD34_primary	H3K4me1	6.01×10^{-8}

Supplementary Table 8. Heritability enrichment of mLOY in hematopoietic lineage cells evaluated by LDSC-SEG.

Name	Coefficient	Coefficient_SE	Coefficient_P_value
MPP	2.2 $x10^{-7}$	4.8 $x10^{-8}$	8.5 $x10^{-7}$
HSC	2.2 $x10^{-7}$	5.2 $x10^{-8}$	3.5 $x10^{-6}$
MEP	1.6 $x10^{-7}$	4.1 $x10^{-8}$	0.000034
CMP	1.8 $x10^{-7}$	4.6 $x10^{-8}$	0.000042
LMPP	1.9 $x10^{-7}$	5.5 $x10^{-8}$	0.00015
GMP	1.8 $x10^{-7}$	5.3 $x10^{-8}$	0.00022
CLP	3.1 $x10^{-7}$	9.0 $x10^{-8}$	0.00056
Erythro	2.4 $x10^{-7}$	9.6 $x10^{-8}$	0.0079
Bcell	1.1 $x10^{-7}$	6.2 $x10^{-8}$	0.049
NK	9.5 $x10^{-8}$	5.7 $x10^{-8}$	0.056
Mono	8.8 $x10^{-8}$	7.0 $x10^{-8}$	0.11
CD8	7.3 $x10^{-8}$	6.6 $x10^{-8}$	0.12
CD4	9.8 $x10^{-8}$	9.1 $x10^{-8}$	0.14

SE: standard error

HSC:hematopoietic stem cell, MPP:multipotent progenitor, LMPP:lymphoid-primed multipotent progenitor, CMP:common myeloid progenitor, CLP:common lymphoid progenitor, GMP:granulocyte/macrophage progenitor, MEP:megakaryocyte/erythrocyte progenitor, CD4:CD4+ T cell, CD8: CD8+ T cell, B:B cell, NK:natural killer cell, Mono:monocyte, Erythro:erythrocyte

Supplementary Table 9. Genetic correlation between mLOY and quantitative traits or malignancies.

Category	phenotype	abbreviation	rg	se	p
Hematological	Mean corpuscular hemoglobin concentration	MCHC	0.13	0.072	0.077
	Platelet count	Plt	0.071	0.059	0.23
	Hemoglobin	Hb	0.079	0.067	0.24
	Red blood cell count	RBC	0.057	0.059	0.33
	Hematocrit	Ht	0.06	0.066	0.36
	White blood cell count	WBC	0.039	0.057	0.5
	Monocyte count	Mono	0.05	0.077	0.52
	Mean corpuscular volume	MCV	-0.031	0.05	0.54
	Neutrophil count	Neutro	0.038	0.066	0.56
	Eosinophil count	Eosino	0.042	0.084	0.61
	Lymphocyte count	Lym	-0.032	0.082	0.7
	Basophil count	Baso	-0.012	0.093	0.89
	Mean corpuscular hemoglobin	MCH	-0.0055	0.054	0.92
Liver-related	Aspartate aminotransferase	AST	0.21	0.057	0.0002
	Alanine aminotransferase	ALT	0.097	0.059	0.098
	γ -glutamyl transferase	GGT	0.065	0.049	0.18
	Zinc sulfate turbidity test	ZTT	0.15	0.14	0.28
	Alkaline phosphatase	ALP	0.022	0.059	0.7
	Total bilirubin	TBill	-0.014	0.074	0.85
Metabolic	Blood sugar	BS	0.15	0.08	0.053
	Hemoglobin A1c	HbA1c	0.12	0.079	0.14

	Total cholesterol	TC	-0.081	0.07	0.25
	High-density-lipoprotein cholesterol	HDL-C	0.056	0.063	0.37
	Triglyceride	TG	-0.038	0.05	0.45
	Low-density-lipoprotein cholesterol	LDL-C	0.022	0.081	0.78
Protein	Non-albumin protein	NAP	0.094	0.067	0.16
	Total protein	TP	0.1	0.072	0.16
	Albumin/globulin ratio	AG	-0.074	0.061	0.22
	Albumin	Alb	0.054	0.072	0.45
Kidney-related	Estimated glomerular filtration rate	eGFR	0.081	0.059	0.17
	Serum creatinine	sCr	-0.066	0.06	0.28
	Blood urea nitrogen	BUN	-0.06	0.058	0.3
	Uric acid	UA	-0.034	0.048	0.48
Electrolyte	Chloride	Cl	-0.11	0.067	0.1
	Calcium	Ca	-0.14	0.089	0.11
	Sodium	Na	-0.1	0.064	0.11
	Phosphorus	P	0.16	0.11	0.13
	Potassium	K	-0.09	0.062	0.14
Other biochemical	Activated partial thromboplastin time	APTT	0.16	0.12	0.17
	Creatine kinase	CK	0.043	0.066	0.51
	C-reactive protein	CRP	-0.026	0.14	0.85
	Fibrinogen	Fbg	-0.031	0.26	0.91
	Prothrombin time	PT	-0.0083	0.1	0.94

	Lactate dehydrogenase	LDH	-0.0028	0.09	0.98
Cancers					
	lung cancer		-0.3	0.39	0.35
	esophagus cancer		-0.204	0.06	0.11
	Endometrial cancer		-0.131	0.34	0.14
	hepatocellular carcinoma		-0.087	0.68	0.21
	cervical cancer		-0.059	0.59	0.11
	prostate cancer		-0.001	0.99	0.12
	colorectal cancer		-0.037	0.72	0.1
	breast cancer		0.033	0.78	0.12
	ovarian cancer		0.078	0.59	0.14
	hematopoietic malignancy		0.098	0.7	0.26
	gastric cancer		0.161	0.18	0.12
	pancreatic cancer		0.18	0.44	0.23

Traits are sorted according to categories and p-values. Definition of categories are based on the previous study. Positive correlation indicates that genetic components increasing QTL trait tend to shorten Y signals. Rg: genetic correlation coefficient, se:standard error

Supplementary Table 10. Significant pathways of mLOY indicated by PASCAL.

Name	P value
REACTOME_CELL_CYCLE	$6.5x10^{-8}$
REACTOME_CELL_CYCLE_MITOTIC	$4.4\ x10^{-7}$
KEGG_CELL_CYCLE	$2.2\ x10^{-6}$

Pathways exceeding significant level (0.05/1077) are indicated.

Supplementary Table 11. Disease status used as covariates in the analysis

Disease	Number of subjects
Diabetes Mellitus	23,445
Hyperlipidemia	20,837
Myocardical infarction	9,775
Cerebral infarcation	9,578
Stable angena pectoris	9,564
Atrial fibrillation	9,449
Catalact	8,342
Prostate Cancer	4,531
urolithiasis	4,522
Heart Failure	4,346
Gastric Cancer	4,199
Colon Cancer	3,777
Asthma	3,675
Unstabel angena pectoris	2,949
HCV hepatitis	2,827
Hay Fever	2,266
Emphysema	2,215
Lung Cancer	2,205
Glaucoma	2,065
Arteriosclerosis obliterans	1,922
Atopic dermatitis	1,374
Gum disease	1,272
Epilepsy	1,123
Liver cancer	1,020

Esophagus cancer	971
Liver cirrhosis	911
Intracranial Aneurysm	862
HBV hepatitis	784
Rheumatoid arthritis	767
Hematopoietic malignancy	591
Graves disease	551
Nephrosis	513

1. Okada Y*, et al.* Deep whole-genome sequencing reveals recent selection signatures linked to evolution and disease risk of Japanese. *Nature communications* **9**, 1631 (2018).

2. Howie BN, Donnelly P, Marchini J. A flexible and accurate genotype imputation method for the next generation of genome-wide association studies. *PLoS genetics* **5**, e1000529 (2009).

3. Delaneau O, Marchini J, Genomes Project C, Genomes Project C. Integrating sequence and array data to create an improved 1000 Genomes Project haplotype reference panel. *Nature communications* **5**, 3934 (2014).

4. Akiyama M*, et al.* Characterizing rare and low-frequency height-associated variants in the Japanese population. *Nature communications (in press)*.

5. Hujoel MLA, Gazal S, Hormozdiari F, van de Geijn B, Price AL. Disease Heritability Enrichment of Regulatory Elements Is Concentrated in Elements with Ancient Sequence Age and Conserved Function across Species. *Am J Hum Genet* **104**, 611-624 (2019).

6. Finucane HK*, et al.* Partitioning heritability by functional annotation using genome-wide association summary statistics. *Nat Genet* **47**, 1228-1235 (2015).

7. Kanai M*, et al.* Genetic analysis of quantitative traits in the Japanese population links cell types to complex human diseases. *Nat Genet* **50**, 390-400 (2018).

8. Forsberg LA*, et al.* Mosaic loss of chromosome Y in peripheral blood is associated with shorter survival and higher risk of cancer. *Nat Genet* **46**, 624-628 (2014).

9. Wright DJ*, et al.* Genetic variants associated with mosaic Y chromosome loss highlight cell cycle genes and overlap with cancer susceptibility. *Nat Genet* **49**, 674-679 (2017).
